# Targeting inhibition of HMGB3 protein transposition in cardiac mitochondria to improve myocardial ischemia reperfusion injury

**DOI:** 10.1186/s12967-026-07920-w

**Published:** 2026-05-20

**Authors:** Chunhua Ma, Jing Wang, Xiaoyong Peng, Tao Li, Liangming Liu

**Affiliations:** 1 Nanotechnology Research Center of Traditional Chinese Medicine, The Eighth Clinical Medical College of Guangzhou University of Chinese Medicine, Foshan Hospital of Traditional Chinese Medicine, No.6 Qinren Road, Chancheng District, Foshan, Guangdong 528000 China; 2 State Key Laboratory of Trauma, Burns and Combined Injury, Shock and Tranfusion Research Department of Army Medical Center, Army Medical University, Chongqing, 400042 P. R. China; 3 School of Biology and Food Engineering, Institute of Pharmaceutical Pharmacology Research Center, Suzhou University, Suzhou, Anhui China

**Keywords:** Proteomic, Myocardial infarction, Mitochondrial targeting, siRNA

## Abstract

**Background:**

Myocardial infarction (MI), as a manifestation of acute myocardial ischemia of coronary heart disease. The aim of this study is to search for the new targets and intervention strategy for MI.

**Methods:**

1242 MI patients and healthy people were enrolled from four medical centers. Proteomics technique was used to analyze the differential expressed proteins (DEPs) between AMI patients and healthy people. The effect and intervention effect of DEPs were investigated with gene knockout mice hypoxia/reoxygenation (H/R) cell and mitochondrial targeted siRNA. 4628 proteins and 365 DEPs were identified in MIT.

**Results:**

High mobility group protein B3 (HMGB3) was the most obvious DEP, which was up-regulated in AMI patients compared with healthy people. Knockout or silence of HMGB3 canceled the effects of HMGB3. Triphenylphosphine (TPP)-HMGB3-siRNA (TPP-MIT-HMGB3-siRNA) could directly target myocardial mitochondria and intervene the myocardial dysfunction caused by HMGB3.

**Conclusions:**

HMGB3 is an important biomarker and plays a critical role in acute myocardial ischemia/reperfusion (I/R) injury. TPP-MIT-HMGB3-siRNA is a powerful mean to intervene acute myocardial ischemic injury.

**Supplementary Information:**

The online version contains supplementary material available at 10.1186/s12967-026-07920-w.

## Introduction

As an acute ischemic manifestation of coronary heart disease, acute myocardial infarction (AMI) is a main cause of adult death [1]. AMI is the most dangerous manifestation of coronary atherosclerotic heart disease [2]. The incidence rate of AMI is increasing year by year over the world, which seriously affects people’s health and medical expenses [3, 4]. When coronary artery insufficiency, myocardial ischemia, hypoxia, or necrosis may lead to ventricular wall movement or arrhythmia, thus resulting in cardiac ejection dysfunction. Long-term ischemia or hypoxia after AMI leads to irreversible death of myocardial cells and negative myocardial remodeling reaction [5]. Therefore, it is urgent to find biomarkers for acute myocardial ischemia such as AMI and intervene the pathophysiological process according to biomarkers.

There are many hypotheses about the etiology and pathogenesis of AMI, but the exact mechanism is not clear yet. AMI is a disease with complex phenotype, which is affected by the complex interactions and mutual relations among genes, genomes, protein groups and environmental factors [6]. Proteomics is a scientific tradition to study the composition and function of all protein in certain tissues. In recent years, proteomics has been used in AMI research [7]. Although these studies provide some valuable theories and basis for the pathogenesis of AMI, but the pathogenesis of AMI is still unclear. The treatment principle of acute myocardial infarction is to restore myocardial blood perfusion as soon as possible, reduce myocardial ischemia, improve cardiac function, and actively manage complications. With the continuous development of medical technology, the treatment of acute myocardial infarction has also made progress, improved survival rates and significantly improving the prognosis of acute myocardial infarction [8]. Although the development of direct Percutaneous coronary intervention and Cardiac surgery bypass surgery has effectively reduced the mortality of acute myocardial infarction in the past three decades, the recurrence rate of postoperative myocardial infarction and the incidence rate of chronic heart failure have gradually increased. Some patients who have undergone thrombolysis and vascular reconstruction continue to decline in cardiac function, greatly reducing the quality of life of patients, even endangering life.

Mitochondrial damage is one of the main causes of myocardial cell necrosis. ROS in myocardial cells is mainly synthesized in mitochondria. Mitochondrial dysfunction after AMI will lead to a large amount of ROS synthesis and release into cytoplasm, thus aggravating myocardial injury. Therefore, Drug design targeting myocardial mitochondria has become a research hotspot in the cardiovascular field. The bilayer phospholipid structure of mitochondria presents a negative Membrane potential [9]. Using Lipophilicity cations, such as triphenylphosphine (TPP), dextrinium (DQA), heptamethyl base dyes, cyclic metallized iridium complexes and Rhodamine derivatives, modifying drug molecules has become the most effective strategy for drug delivery to mitochondria. Among them, TPP has relatively low biological toxicity [8], and polymer nano micelles carrying TPP have become the main candidate for mitochondrial targeted drug delivery. Therefore, the present study is aimed to: (1) search for the differentially expressed proteins (DEPs) for acute myocardial ischemia. (2) identify the functional biomarkers (proteins). (3) confirm the protective effect of mitochondrial targeting DEPs-siRNA for acute myocardial ischemia.

## Materials and methods

### Reagents

L-lactide, dichloromethane, chloroform, absolute ether, cholamine were purchased from Sinopharm Group Chemical Reagent Co., Ltd. 3-bromopropionic acid and TLR4 inhibitor (TAK-242) were obtained from Shanghai Aladdin Biochemical Technology Co., Ltd. HMGB3 antibody (KMA029011R) was obtained from Wenzhou Kemiao Biological Co., Ltd. Tumor necrosis factor (TNF)-α, interleukin (IL)-6, IL-1β Enzyme linked immunosorbent assay (ELISA) kits were obtained from Elabscience (Wuhan, China). All antibodies were purchased from Cell Signaling Technology. H9c2 cells were purchased from Shanghai Institute of Biological Cells.

### Animals

WT mice (ICR, 6 weeks, 16–18 g), HMGB3 (ICR, HMGB3^−/−^, 6 weeks, 16–18 g) were obtained from the Cyagen Biosciences Inc. All animal operations were approved by the Research Council and Animal Care and Use Committee of Animal Center of Army Medical Center.

### AMI patients and proteomics

**AMI patients.** In this study, 1242 patients with acute myocardial infarction (AMI) [621 patients with ST segment elevation myocardial infarction (STEMI) and 621 patients with non ST segment elevation myocardial infarction (NSTEMI)] and 60 healthy people (volunteer) were selected from the Affiliated Hospital of Nanjing University of Traditional Chinese Medicine, Nanjing Hospital of Traditional Chinese Medicine, Hospital of Integrated Traditional Chinese and Western Medicine, and the Second Affiliated Hospital of Nanjing University of Traditional Chinese Medicine from January 2017 to January 2019. All the selected patients came from inpatients in ICU and CCU, and this study was approved by the Hospital ethics committee and all enrolled patients signed an informed consent. All patients with AMI were diagnosed according to the diagnostic criteria outlined in the European Society of Cardiology (ESC) guidelines in 2019 is admission within 24 h of chest pain. General information of patients was seen in Table 1.

**Table 1:** General information of patients

	Control (*n* = 60)	NSTEMI (621)	STEMI (621)	*P* value
Age(year)	46.32 ± 14.51	62.16 ± 9.65	59.31 ± 9.29	0.068
Gender (Male, 100%)	39 (65.00%)	336 (54.10%)	317(51.04%)	0.581
Smoking history (%)	32(60.00%)	305(49.11%)	301(48.47%)	0.752
History of hypertension (%)	26(43.33%)	137(22.06%)	131(21.09%)6	0.281
History of diabetes (%)	4(6.70%)	12(1.93%)	14(2.25%)	0.473
Family history (%)	0(0.00%)	5(0.80%)	7(1.12%)	0.592
Anterior	0(0.00%)	417(67.14%)	432(69.56%)	0.261
Septal	0(0.00%)	109(17.55%)	114(18.35%)	0.318
Inferior	0(0.00%)	95(15.29%)	75(12.07%)	0.227

**Exclusion criteria.** (1) The patient had previous myocardial infarction and underwent coronary intervention (PCI) or coronary artery bypass grafting (CABG); (2) The patient has cardiogenic shock, severe heart failure, arrhythmia, myocarditis, infective endocarditis, severe hemodynamic abnormality or valvular disease and other cardiovascular diseases; (3) The patient has serious endocrine and metabolic disorders; (4) The patient is in the active stage of infectious diseases such as tuberculosis, pulmonary infection, viral hepatitis and sepsis; (5) The patient has severe liver and kidney dysfunction; (6) Patients have blood, rheumatic immunity, tumor diseases, etc., and use glucocorticoids, immunosuppressants, chemotherapy drugs.

#### Detection of myocardial markers

**Cardiac troponin (cTn).** cIn was detected by chemiluminescence immunoassay according to the kit instructions.

**Creatine kinase (CK) and creatine kinase isoenzymes (CK-MB).** CK was determined by creatine kinase substrate method and CK-MB was determined by immunosuppression method.

**Serum alanine transaminase (ALT) and aspartate aminotransferase (AST).** Alanine substrate method was used to detect ALT and aspartate substrate method was used to detect AST.

**Serum creatinine (Cr) and urea nitrogen (UN).** Cr was detected by sarcosine oxidase method and UN was detected by urease-glutamate dehydrogenase method.

**Left ventricular end diastolic diameter (LVEDD)**,** left ventricular ejection fraction (LVEF) and cardiac output (CO).** PHILIPS IE33 ultrasonic diagnostic instrument was used, and the probe frequency was 2.5–3.5 MHz All patients’ cardiac morphological structure and hemodynamic indexes, including changes of LVEDD, LVEF and CO, were measured by the same physician to evaluate the changes of cardiac function 24 h after admission.

#### Proteomics of AMI

**Extraction of protein and enzymatic hydrolysis of peptides.** Proteins in serum tissue were extracted by STD (4% SDS, 100 mM Tris/Hcl PH = 7.6, 0.1 M DTT) cracking method. Then BCA was used to quantify the proteins and samples were digested with trypsin. C_18_ cartridge was used to desalt the hydrolyzed peptide, and the peptide was quantified.

**iTRAQ labeled protein samples.** 100 µg peptide fragment was labeled with iTRAQ according to the instructions of the kit.

**Fractionation of peptides by strong cation exchange chromatography.** iTRAQ labeled peptides were mixed and graded with AKTA purifier 100. The mobile phase is divided into A (10 mM KH2PO4, 25% ACN PH = 3.0) and B (10 mM KH2PO4, 500 mM KCL, 25% ACN PH = 3.0). The mobile phase gradient is as follows: 0% B 25 min, 0%-10% 25–32 min, 10%-20% B 32–42 min, 20%-45% B 42–47 min, 45%-100% B, 47–52 min, 100%B 52–60 min.

**LC-MS/MS data acquisition.** Capillary high performance liquid chromatography: nano-HPLC Easy NLC system was used for separation. The loading column was Thermo scientific EASY C18 (2 cm×100 μm, 5 μm) and the analysis column was Thermo scientific EASYC18 (75 μm×100 mm, 3 μm), with a flow rate of 300nL/min. Gradient elution of buffer A (0.1% formic acid-water solution)-B (0.1% formic acid − 84% acetonitrile water solution): 0–55 min, 0%-40% B; 55–58 min, 40%-100%B; 58 ~ 60 min, 100%B.

### Synthesis of mitochondrial targeting materials

**Synthesis of carboxyamyl triphenyl bromide scale (TPP-COOH).** Triphenyl phosphine (TPP, 10 mmol) and 6-bromohexanoic acid (10.5 mol) were dissolved in anhydrous acetonitrile and refluxed for 16 h under the protection of nitrogen. The reactant was recrystallized to obtain TPP-COOH.

**Synthesis of TPP-polymine (PEI) polymer.** TPP-COOH (2 mmol), N, N-dicyclohexylcarbodiimide (2.4 mmol) and N-monohydroxy succinimide (NHS, 2. 4 mmo1) were dissolved in anhydrous DMSO 10 m L and reacted at room temperature for 12 h, then PEI (1 mmol) was added to continue the reaction at room temperature. 12 h later, the reaction solution was transferred to a dialysis bag (with a cut-off molecular weight of 1000), and dialyzed with DMSO for 24 h and deionized water for 48 h. The dialysate was freeze-dried to obtain intermediate products (TPP-PEI).

**Combination of TPP-PEI-siRNA polymer.** In RNA-free water, a certain amount of siRNA (HMGB3 siRNA, sequence of siRNA can be seen in Table 1) was mixed with TPP-PEI solution by gentle pipetting. Then, the mixture was vortexed for 30 s, and then kept still at room temperature for 30 min to form a TPP-PEI/siRNA complex.

**Structural phenotype.** TPP-COOH and TPP-PEI were identified by ^1^HNMR and infrared spectrum.

**Gel migration retardation analysis of siRNA complexation.** The binding between TTP-PEI and siRNA was detected by agarose gel electrophoresis. TTP-PEI-siRNA was treated with buffers with different pH values for electrophoresis.

#### The binding of TTP-PEI-siRNA to H9c cells was observed by projection electron microscope (CLSM)

The binding between TTP-PEI-siRNA and H9c cells was observed by CLSM. This detection has the potential to judge the siRNA released by TTP-PEI-siRNA.

### Animal model of myocardial ischemia reperfusion(I/R) and related analysis

**Establish of I/R model.** Wild type mice and KO mice were divided into the following groups: (1) WT mice (Ctr, sham group), (2) WT mice + I/R (I/R, I/R group), (3) I/R + TTP-PEI-HMGB3 siRNA (HK, 1 ng/kg) group, (4) HMGB3 KO mice + IR + TTP-PEI-siRNA (TPS, 5 ng/kg) group. Ischemia-reperfusion (I/R) model was established by coronary artery ligation. The specific operation was as follows: the mice were anesthetized by intraperitoneal injection of pentobarbital sodium solution 50 mg/kg, the skin between the second and fifth ribs of the left chest of the mice was cut open, the subcutaneous tissue was separated, the second and third ribs were sawn off, the heart was exposed, the coronary artery was ligated and maintained 30 min and then the ligation was removed. Then the skin was sterilized and sutured. The sham operation group only operated without ligation and reperfusion. During the ischemia-reperfusion period, the animal body temperature was maintained by lamp burning. TTP-PEI-siRNA was given to mice by intravenous delivery during reperfusion, it is injected through caudal vein. After 24 h of the intervention, the hemodynamics was hemodynamics was detected by small animal hemodynamic monitoring system and electrocardiogram of animals were tested.

**Acquisition of mouse serum and heart samples.** The mice were euthanized by inhaling isoflurane (35 mg/kg), and the whole blood was centrifuged at 4℃, 3000r/min, and the supernatant was obtained. The serum was used for CK, CK-MB, cTn, IL-1β, IL-6 and TNF-α detection. At the same time, the myocardial tissue in infarcted area was fixed with formaldehyde for histopathology, and other hearts were frozen in a refrigerator at -80℃ for mechanism research.

### In vitro model and analysis of hypoxia and reoxygenation (H/R)

**H9c2 cells culture.** H9c2 cells were inoculated in serum-free complete F12 medium in 1 × 10 ^5^/60 mm plates, and cultured in 5% CO2 incubator at 37℃.

**Knockdown of HMGB3 in H9c2.** H9c2 cells were transfected with HMGB3 and TLR4 (Shanghai GenePharma, China) or negative control (NC) siRNA (Shanghai GenePharma, China) as the method mentioned in the instructions. The sequence of HMGB3 and TLR4 was listed in the Table 2. Lipofectamine 2000 was used for transient siRNA transfections (Invitrogen) according to the manufacturer’s recommendation. A complex of 0.5 mL Opti-MEM medium and 6 µL of Lipofectamine 2000 containing 50 nM HMGB3 and TLR4, or 50 nM NC were premixed per well in a 6-well plate.

**Table 2:** HMGB3 siRNA, TLR4 siRNA and STAT3 siRNA sequences

Gene	Upstream primer sequence (5 ‘-3 ‘)	Upstream primer sequence (5 ‘-3 ‘)
HMGB3	GACCAGCTA AGGGAGCWAA	ACAGGAAGAATCCAGACGGT
TLR4	TTTATTCAGAGCCGTTGG	GGCTATCTGTGAGCGTGT
STAT3	CCGUGGAACCAUACACAAA	UUUGUGUAUGGUUCCACGG

**Build of H/R in H9c2 cells.** The normal cells, transfected cells (HMGB3 siRNA) and were put into an anoxic incubator. The model construction time was 2 h after hypoxia and 4 h after reoxygenation. During reoxygenation, F12 medium without serum was used to replace glucose-free medium, and the culture was continued for 4 h in a conventional 5% CO2 cell incubator. In the reoxygenation stage, cells were incubated with TTP-PEI-siRNA (5 nM) for 4 h. The supernatant was used for CK, CK-MB, cTn detection. The cells were used for mechanism research.

**Culture solution collection and analysis.** Culture solution from H/R-stimulated H9c2, HMGB3 knocked down H9c2 cells was centrifuged at 3000 r/m, 4 ℃ for 10 min and the supernatant was collected for CK, CK-MB and cTn detection.

**H9c2 collection and analysis.** H/R-stimulated cells, HMGB3 knocked down H9c2 cells were collected. Part of the cells was used to extract proteins, and the protein concentration was determined by bicinchoninic acid assay (BCA) and for mechanism research. The other part of cells was used to determine the CK, CK-MB and cTn.

### Interaction between HMGB3 and TLR4

#### Cell transfection

H9c2 cells were cultured in DMEM medium containing 10% bovine serum. 24 h before transfection, the cells were inoculated in 12 well plates with the corresponding medium containing 10% neonatal bovine serum without double antibodies, and the inoculation amount was subject to the cell density reaching 90% at the time of transfection. DNA was diluted with 80 µL medium without double antibody and fetal bovine serum. 2.5 µL of Lipofectamine 2000 was diluted with the same medium as above, and the two were mixed immediately. After being placed at room temperature for 20 min, it was added to a 12 well plate containing 0.8 mL medium and 10% newborn bovine serum, and the medium was changed after 4 h.

#### Luciferase and β-Galactosidase (β-Gal) activity determination

Basically, according to the instructions of the kit. 24 h after transfection, the cells were centrifuged (12000 r/min, 10 min, 4 ℃), and the supernatant was taken to determine the luciferase activity. Above 10 µL supernatant was added to 450 µL buffer contains β-mercaptoethanol (2.7 mL/L), 100 µL 0.4% o-nitrobenzene-β-D-galactoside solution was added and kept at 37 ℃ until light yellow appears, 250 µL1mol/L Na_2_CO_3_ was added, the reaction was terminated, and the value of sample a was measured at the wavelength of 420 nm.

#### To detect the extracellular interaction between HMGB3 and TLR4

PET28a (+) -HMGB3 and pGEX kg TLR4 plasmids were constructed and translated in vitro by reticulocyte system. His HMGB3 fusion protein with radioisotope ^35^S was obtained. Gst-TLR4 fusion protein was obtained by protein purification technique. With 11 µL His-HMGB3 in vitro translation products and 13 µL agarose beads with Gst-TLR4 fusion protein for binding reaction, which was named group 1. At the same time, the negative control group was set as the group 2, and the sample contains 11µL His-HMGB3 in vitro translation products ＋ 13 µL agarose beads bound to GST protein. After the reaction was completed, run the page adhesive for autoradiographic identification, autoradiographic identification with PAGE adhesive.

#### To detect the interaction between HMGB3 and TLR4 in H9c2 cells

H9c2 cells were inoculated in four 6 cm plates. When the cell density reached 90%, the culture medium was replaced 1 h before transfection, with a volume of 1.5 mL. Specific grouping and plasmid dosage were as follows: (1) PcDNA3-flag unstimulated group, PcDNA3-flag (0.2 µg/dis)＋pcDNA3-HMGB3 (0.8 µg/dish); (2) PcDNA3-flag stimulated group, PcDNA3-flag (0.2 µg/dis)＋pcDNA3-HMGB3 (0.8 µg/dish); (3) Co- transfer pcDNA3 flag-TLR4 unstimulated group, PcDNA3-flag-TLR4 (0.2 µg/dis) ＋ pcDNA3-HMGB3 (0.8 µg/dish); (4) Co-transfer pcDNA3 flag-TLR4 stimulated group, PcDNA3-flag-TLR4 (0.2 µg/dis) ＋ pcDNA3-HMGB3 (0.8 µg/dish). After transfection, the new culture medium was replaced 4 h later, and the cells were collected 24 h later for immune sedimentation test.

#### GST pulldown experiment

11µL His-HMGB3 in vitro translation products and 13 µL combined with GST-agarose beads of TLR4 fusion protein were used for binding reaction. At the same time, the negative control group was set up. The in vitro translation product of His-HMGB3 was combined with the agarose beads bound with GST protein, and was incubated overnight at 4 ℃. After the reaction, PAGE-gel was run, and the sequence of electrophoretic loading was His-HMGB3 protein control, binding reaction negative control group and binding reaction group. After electrophoresis, drain the gel, press it, and develop it for autoradiographic identification after 3 days.

#### Immunoprecipitation (CO-PI) test

24 h after transfection, the culture dish was taken out and the cells were collected. The supernatant was retained after ultrasonic fragmentation. Of which, 30 µL was extracted from the supernatant reserved for electrophoresis input, the rest is about 420 µL supernatant was used for IP experiment. 15µL Flag beads was added to each sample. After settling, the sample was added to the loading buffer, fully mixed, boiled and centrifuged. 10 µL sample was for western blot analysis. The sequence of electrophoretic loading was input 1, input 2, input 3, input 4, group 1, group 2, group 3, group 4.

### Verify the effects of TLR4 inhibitor (TAK-242) and TLR4 siRNA on STAT3 in I/R and H/R models

#### In I/R model

The mice were divided into the following groups: (1) sham mice (Ctr group), (2) control mice + I/R group (I/R), (3) I/R + HMGB3 KO, (4) I/R mice + TAK-242 (5 mg/kg) group, (5) I/R + HMGB3 KO + TAK-242 (5 mg/kg) group. Ischemia-reperfusion (I/R) model was established by coronary artery ligation. The specific operation was as follows: the mice were anesthetized by intraperitoneal injection of pentobarbital sodium solution 50 mg/kg, the skin between the second and fifth ribs of the left chest of the mice was cut open, the subcutaneous tissue was separated, the second and third ribs were sawn off, the heart was exposed, the coronary artery was ligated and maintained 30 min and then the ligation was removed. Then the skin was sterilized and sutured. The sham operation group only operated without ligation and reperfusion. During the ischemia-reperfusion period, the animal body temperature was maintained by lamp burning. TAK-242 (5 mg/kg) was given to mice by intravenous delivery during reperfusion, it is injected through caudal vein. After 24 h of the intervention, the hemodynamics was hemodynamics was detected by small animal hemodynamic monitoring system and electrocardiogram of animals were tested.

#### In H/R model

The model construction time was 2 h after hypoxia and 4 h after reoxygenation. During reoxygenation, F12 medium without serum was used to replace glucose-free medium, and the culture was continued for 4 h in a conventional 5% CO2 cell incubator. In the reoxygenation stage, cells were incubated with HMGB3 siRNA and TLR4 siRNA (5 nM) for 4 h. The supernatant was used for CK, CK-MB, cTn detection. The cells were used for mechanism research.

### Mitochondrial function

#### Mitochondrial respiration

The oxygen consumption rate (OCR) was measured using a 24-well XFe plate (Seahorse, Agilent Cell Analysis technology, USA). H9C2 cells were seeded at a density of 1 × 10^4^ per well, and put the cell plate in the super-clean bench for 1 h to make the cells settle naturally. Then the cell plate was put back into the cell incubator overnight to make the cells adhere to the wall. When the cell confluence reaches 70–80%, HMGB3 sirRNA (10 µmol/L) or TPS (10 µmol/L) were added to treat cells for 12 h, 24 h and 48 h. Before detection, H9C2 cells were added LPS. Then the basic assay medium contains 2.5µM glucose and 2mM glutamine was used to culture cells for 50 min. Then 2µM oligomycin, 1µM FCCP, and 0.5µM rotenone/antimycin A were performed sequentially. The OCR was measured by an extracellular flux analyzer under the mitochondrial stress test condition.

#### Mitochondrial morphology and mitochondrial membrane potential

The H9C2 cells treated above were washed with 4 ℃ pre cooled PBS (pH 7.2) for 3 times, 1.0 ml PBS, 10 µL 2.5 mmol/l JC-1 were added respectively, so that the final concentration was 25 µmol/L. They were incubated in a 37 ℃ 5% CO2 incubator for 20 min in the dark, washed with PBS for 3 times, and detected and analyzed by laser confocal scanning microscopy.

### Experimental correlation methodology

**Coronary angiography.** At first, the AMI patient was diagnosed with color Doppler Siemens ACUSON S2000 ultrasonic diagnostic instrument. During the examination, the probe frequency was set to 2.5 MHz, and the patient was kept the left lateral position, and then the heart of AMI patient was examined in all directions.

**Electrocardiogram examination.** A 12-lead electrocardiograph produced by Japan Optoelectronic Co., Ltd. was used to carry out resting electrocardiogram examination on all patients and animals, and the changes of ST segment, abnormal T wave and heart rate in each lead were scanned, and then the scanning results were diagnosed by more than two diagnosticians with experienced diagnosis experience.

**Hemodynamics.** Heart rate (HR), left ventricular diastolic pressure (LVDP) at the end of reperfusion were collected by Powerlab/8SP data acquisition and analysis system. LVDP, left ventricular end diastolic pressure (LVEDP) and maximum rate of rise and fall of left ventricular pressure (+/-dp/dtmax).

**Cell viability.** The H/R normal cells and transfected cells were inoculated in a 96-well plate with 1 × 10^4^ cells per well, and each group had 3 wells. After the above-mentioned different treatments, 10 µL MTT solution was added to each well, and the culture was continued at 37℃ for 4 h. The culture solution was sucked off, and 150 µL DMSO was added to each well, and the absorbance of each well was measured at 490 nm (A).

**CK**,** CK-MB**,** cTn and IL-1β**,** IL-6 and TNF-α detection in serum**,** heart and H9c2 cells.** 100 mg heart tissue of WT and KO mice was homogenized in PBS buffer solution at a ratio of 1:10 under ice bath. The homogenate was centrifuged at 4℃ for 12,000/min for 10 min. The supernatant was collected. The levels of CK, CK-MB, cTn and IL-1β, IL-6 and TNF-α in patient and animal serum and heart were detected. The levels of CK, CK-MB and cTn in H9c2 cells were detected.

**HE staining of heart tissue of I/R mice and KO mice.** After dehydration and paraffin embedding, the fixed heart tissues were cut into paraffin section with a thickness of 5 μm and stained with hematoxylin and eosin (HE) and then observed under a light microscopy (Nikon, Tokyo, Japan) at 200× magnification.

**2**,** 3**,** 5-triphenyltetrazole chloride (TTC) staining.** The flattened heart was placed in 1% TTC solution dissolved in PBS (0.1 mol/L), incubated at 37℃ in the dark for 20 m in, then taken out and placed in 10% neutral formaldehyde (0.1 mol/L PBS). Photographs were collected, and the infarct area was calculated by Photoshop 3 S Extended software. Percentage of infarcted area (%) = infarcted area/total area×100%.

**HMGB3 mRNA level in MIT**,** MNIT by PCR.** MIT and MNIT mRNA were extracted by Trizol method, and the level of HMGB3 mRNA was detected by PCR kit.

**Immunohistochemistry.** The levels of HMGB3 in heart of mice and KO mice were determined by immunohistochemistry. Briefly, the heart tissue was fixed with 4% paraformaldehyde (PFA), embedded in paraffin, and then sectioned. The paraffin sections were dewaxed in xylene and dehydrated ethanol, microwaved in sodium citrate buffer, and washed with PBS. Endogenous peroxidase activity was blocked by 3% hydrogen peroxide for 20 min. Each sample was blocked with 5% goat serum for 20 min and then treated overnight with HMGB3 (1:500) antibodies at 4℃. On the next day, each sample was washed three times with PBS before treated with goat anti-rabbit IgG secondary antibody for 20 min. They were then washed three times with PBS afterwards. Next, samples were stained with 3–3’diaminobenzidine (DAB) and later stained with hematoxylin. After dehydrated and dried, the sections were mounted with neutral gum and observed with microscope.

**Immunofluorescence.** The levels of HMGB3 in H9c2 were evaluated by immunofluorescence. Briefly, H9c2 were washed 3 with PBS, fixed with 4% paraformaldehyde (PFA) for 30 min, and then permeabilized with 0.5% Triton X-100 in PBS for 5 min, blocked with 5% BSA for 1 h. The cells were incubated with the primary antibodies HMGB3 (1:500) overnight at 4℃, and then washed three times with PBS and incubated with goat anti-rabbit IgG (H + L) secondary antibody, Alexa Fluor^®^ 488 conjugate (1:500) for 1 h. After three times washing with PBS and the DAPI was added at room temperature for 5 min. Fluorescence images were taken with fluorescence microscopy.

**Western blotting.** Heart tissues and cells were lysed on ice by adding RIPA lysate and 2 µL protease inhibitor PMSF. After full lysis, they were transferred to centrifuge tube and centrifuged at about 12 000 r/min at 4℃ for 20 min. The supernatant was the total protein extract. The BCA method was used for protein quantification, and then SDS-PAGE gel electrophoresis was carried out, and then 0.22 μm PVDF membrane was used for protein transfer. After 5% skimmed milk powder was sealed at room temperature for 2 h, the primary antibody was incubated at 4℃ overnight, and after washing with TBST buffer, the secondary antibody was incubated at room temperature for 1 h. After washing again, ECL developer was dripped on PVDF membrane and exposed for imaging. Image analysis software Image J was used to detect the gray value of protein bands.

### Statistical analysis

SPSS 23.0 statistical software was used to analyze the data, and the measurement data was expressed by mean ± SD. One-way variance analysis was used to compare the differences of multiple samples, and *P* < 0.05 indicated that the difference was statistically significant.

## Results

### Diagnosis and proteomics in patients with AMI

**Fig. 1:**
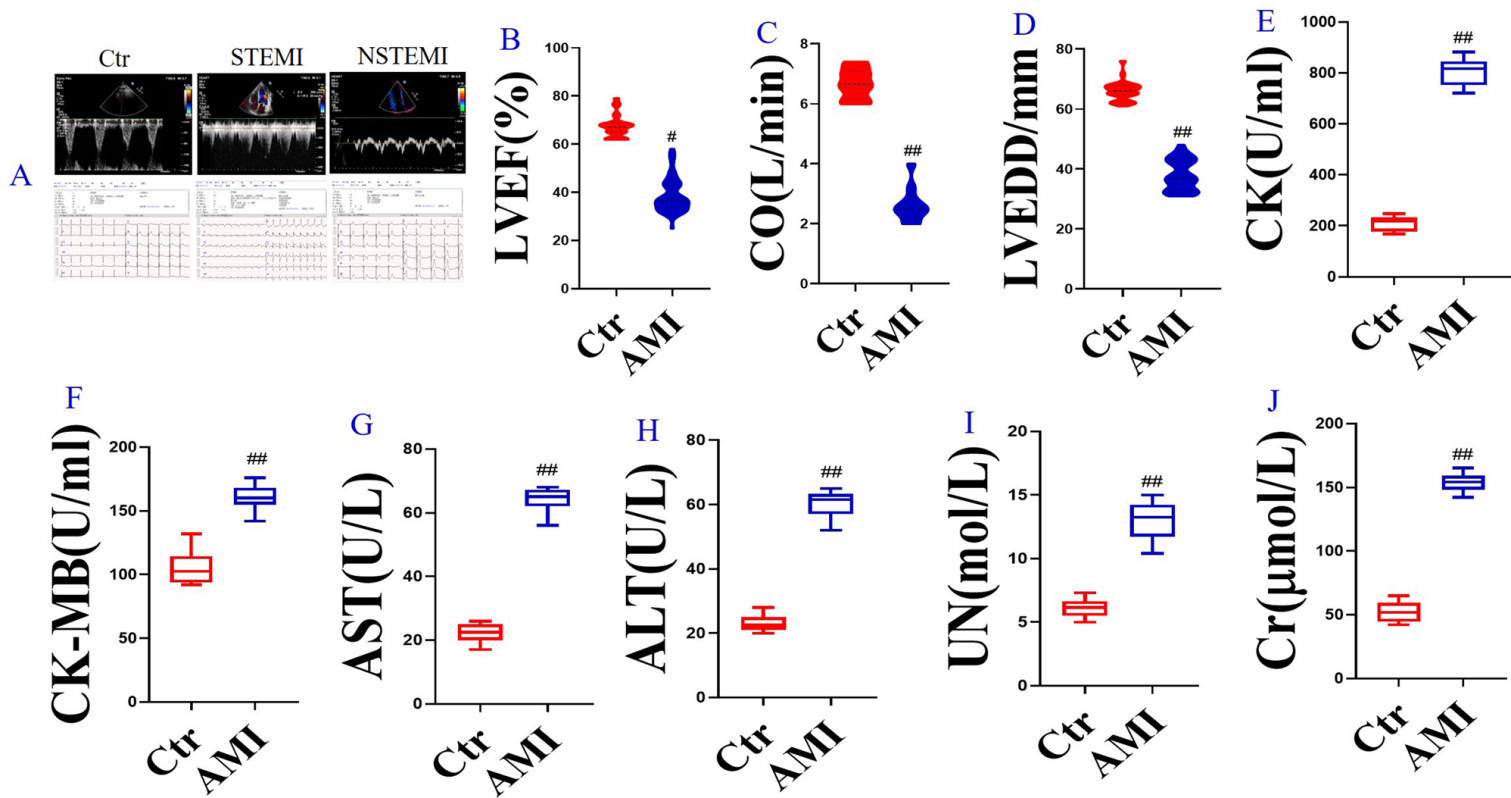
Diagnosis of patients with acute myocardial infarction (AMI). (**A**) Cardiac color doppler ultrasound and Electrocardiogram (EGG) in healthy person and AMI patients; (**B**-**D**) Cardiac color doppler ultrasound analysis of AMI patients: left ventricular end diastolic diameter (LVEDD), left ventricular ejection fraction (LVEF) and cardiac output (CO); (**E**-**J**) CK, CK-MB, cTn, AST, ALT, UN and Cr results in AMI patients. (*n* = 1242 for AMI patients and *n* = 60 for healthy people). All data were presented as mean ± SD. Compared with healthy people: ^##^*P* < 0.01

**Color doppler echocardiography analysis.** As **c**ompared with healthy people, the LVEDD of AMI patients (including STEMI and NSTEMI patients) was significantly decreased, while the CO and LVEF were significantly deceased (Fig. 1A-D).

**Electrocardiogram (EGG) results. As** compared with healthy people, The electrocardiogram showed that the ST segment in STEMI patients was widely increased and the T wave was inverted, accompanied by frequent ventricular extrasystoles and abnormal high-sharp asymmetric T wave. While the ST segment in NSTEMI patients was not increased (Fig. 1A).

**CK**,** CK-MB**,** AST**,** ALT**,** UN**,** and Cr results. As** compared with healthy people, the levels of CK, CK-MB, AST, ALT, UN, and Cr in AMI patients were significantly increased (Fig. 1E-J).

**Fig. 2:**
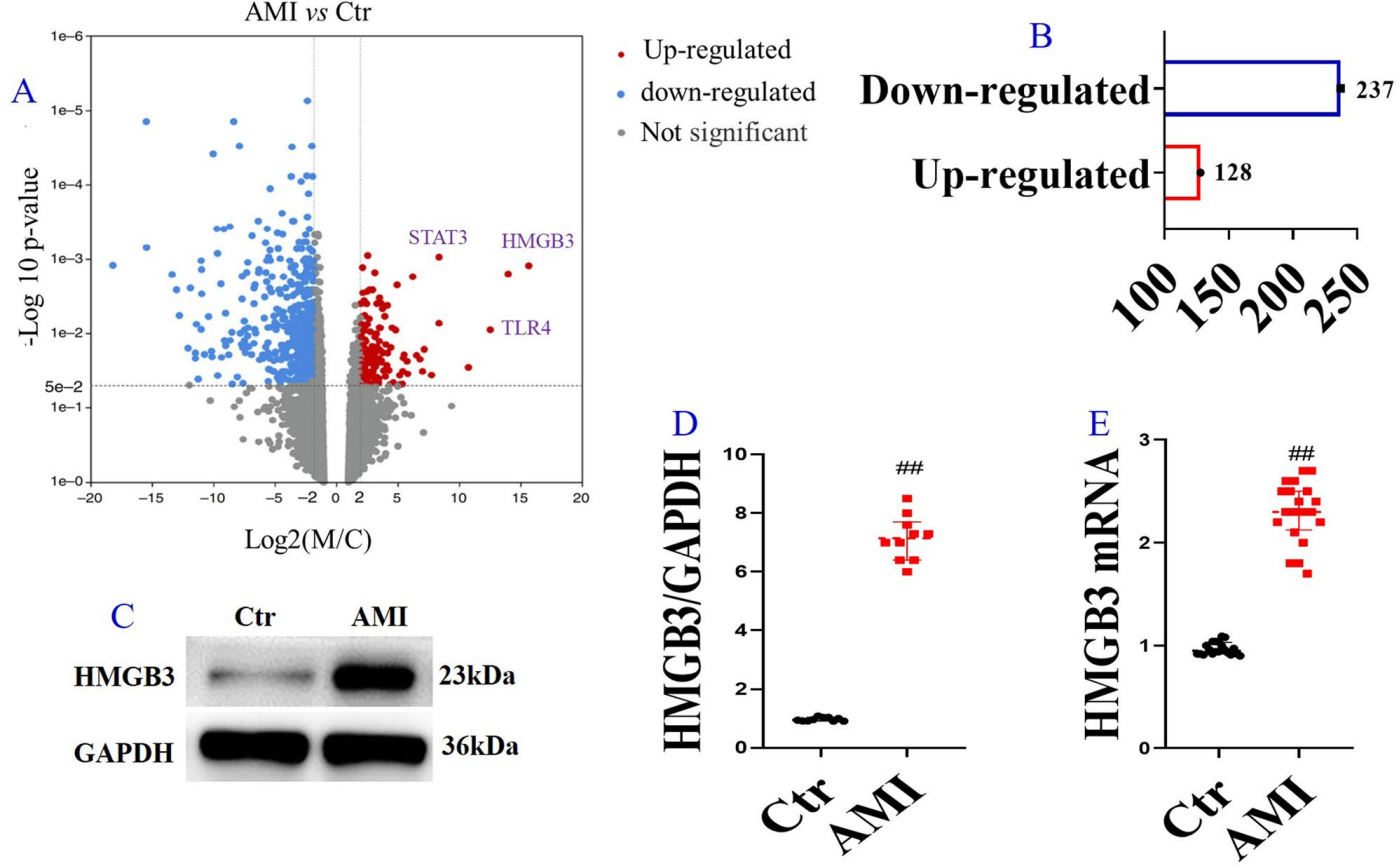
Proteomics analysis. (**A**) Quantitative results of proteins volcano plot; (**B**) Statistics of up and down regulated proteins in healthy person and AMI patients; (**C**-**D**) Western blot and quantitative results for HMGB3, (**E**) PCR results of HMGB3 (M) (*n* = 10). All data were presented as mean ± SD. Compared with healthy people: ^##^*P* < 0.01

**Proteomics results.** A total of 4628 proteins in serum tissue from AMI patients were identified. In order to find the differentially expressed proteins, we formulated the brushing criteria, and brushed the differentially expressed proteins according to the criteria of multiple variation greater than 1.5 and p value less than 0.05. Compared with healthy people, 128 up-regulated differential proteins and 237 down-regulated differential proteins were identified. Quantitative results of proteins volcano plot (Fig. 2A-B) and the information of peptide ion score, mass Error, molecular weight, peptide counts, peptide length, calculated PI, protein ratio, protein sequence coverage. In order to further study differentially expressed proteins, we use cluster analysis to analyze differentially expressed proteins, to obtain the rationality and relative expression quantity of differentially expressed proteins. In order to further study differentially expressed proteins, we use cluster analysis to analyze differentially expressed proteins, so as to obtain the rationality and relative expression quantity of differentially expressed proteins. Cluster analysis identified the differentially expressed protein HMGB3 as one of the proteins with obvious changes.

**The results of HMGB3 mRNA and protein level in serum.** The above results showed that the differential protein HMGB3 was selected by proteomics, and the mRNA and protein levels of HMGB3 were detected by PCR and Western blot. As shown in Fig. 2C-E, as compared with healthy people, the HMGB3 mRNA and protein level was markedly increased in AMI patients. The results showed that the expression of HMGB3 was significantly increased in AMI patients.

### Structural phenotype results of TTP-PEI-mitochondrial targeting siRNA

**Hydrogen spectrum results.** Synthesis diagram of TPP-PEI-siRNA is shown in (supplementary Fig. S1A-C). As shown in supplementary Fig. S1C, TTP-COOH ^1^H NMR features are as δ 12.01 (s, 1H-COOH), 7.91–7.75 (m, 15 H, Ar-H), 3.62–3.53 (m, 2 H, -CH_2_-), 2.17–2.14 (m, 2 H, -CH_2_-), 1.5 (s, 6 H, -CH_2_-). PEI ^1^H NMR features are as δ 2.27–2.25 (m, -NHCH_2_CH_2_-). Supplementary Fig. S1D, showed the characteristic chemical shift of TPP- COOH from 7.75 to 7.91, and the characteristic chemical shift of PEI from 2.27 to 2.55. In the hydrogen spectrum of the intermediate TPP-PEI, there were characteristic shifts of TPP-COOH and PEI, which indicated the successful synthesis of TPP-PEI.

**Infrared spectral characteristics.** As shown in supplementary Fig. S1E, TTP-COOH infrared spectral features are as TPP has benzene ring absorption peaks at 745 cm^− 1^ and 690 cm^− 1^, bromopropionic acid has carbonyl peaks at 1728 cm^− 1^, and both peaks appear in TPP-COOH, indicating that the target product has been successfully synthesized.

**Gel migration retardation results.** As shown in supplementary Fig. S1FS2, gel retardation experiment showed that TPP-PEI polymer combined with siRNA at a ratio of 5:1, and this result showed that TPP-PEI polymer combined with siRNA very well. The particle size range of TPP-PEI/siRNA complex was 92.7–183.2 nm, and the weight ratio of TPP-PEI/siRNA complex was 1/1–50/1. When the weight ratio of TPP-PEI/siRNA complex was greater than 20/1, the zeta potential is positive.

**Stability and biocompatibility results TPP-PEI/siRNA.** Stability was very important for TPP-PEI/siRNA polymer. Therefore, in this experiment, TPP-PEI/siRNA polymer was placed in PBS (PH = 7.4), F12 culture medium and 10% fetal bovine serum (FBS) for 7 days to check the stability of the polymer. As shown in supplementary Fig. S1G-H, it has no effect when placed in these three solutions for 7 days (in the abscissa of Figs. 1 and 4D represents 1:1, 2 represents 5:1, 3 represents 10:1, 4 represents 20:1, 5 represents 40:1, and 6 represents 80:1). Similarly, biocompatibility was also very important for TPP-PEI/siRNA polymers. As shown in supplementary Fig. S1I-J, the TPP-PEI/siRNA polymer from 0 to 20 µg/ml has no effect on the viability of H9c2 cells and does not cause any hemolytic reaction. The results suggest that TPP-PEI/siRNA polymer has good stability and biocompatibility.

**The reaction of TPP-PEI/siRNA polymer to pH value.** After entering the body, pH is one of the biggest challenges for the stability of TPP-PEI/siRNA polymers. Therefore, the TPP-PEI/siRNA polymer was put in solutions with different pH values for different times to judge the sensitivity of the polymers to pH values. As shown in supplementary Fig. S1K-N, the potential of the polymer changed sharply from − 6.8mv (pH = 7.4) to + 35mv (pH = 4.5), and the potential of the TPP-PEI/siRNA polymer did not change after being placed for 6 h under the physiological condition of pH = 7.4, this result indicated that the TPP-PEI/siRNA polymer was relatively stable under the physiological condition of pH.

### The role of HMGB3 in myocardial I/R injury

#### In animal experiments

**Fig. 3:**
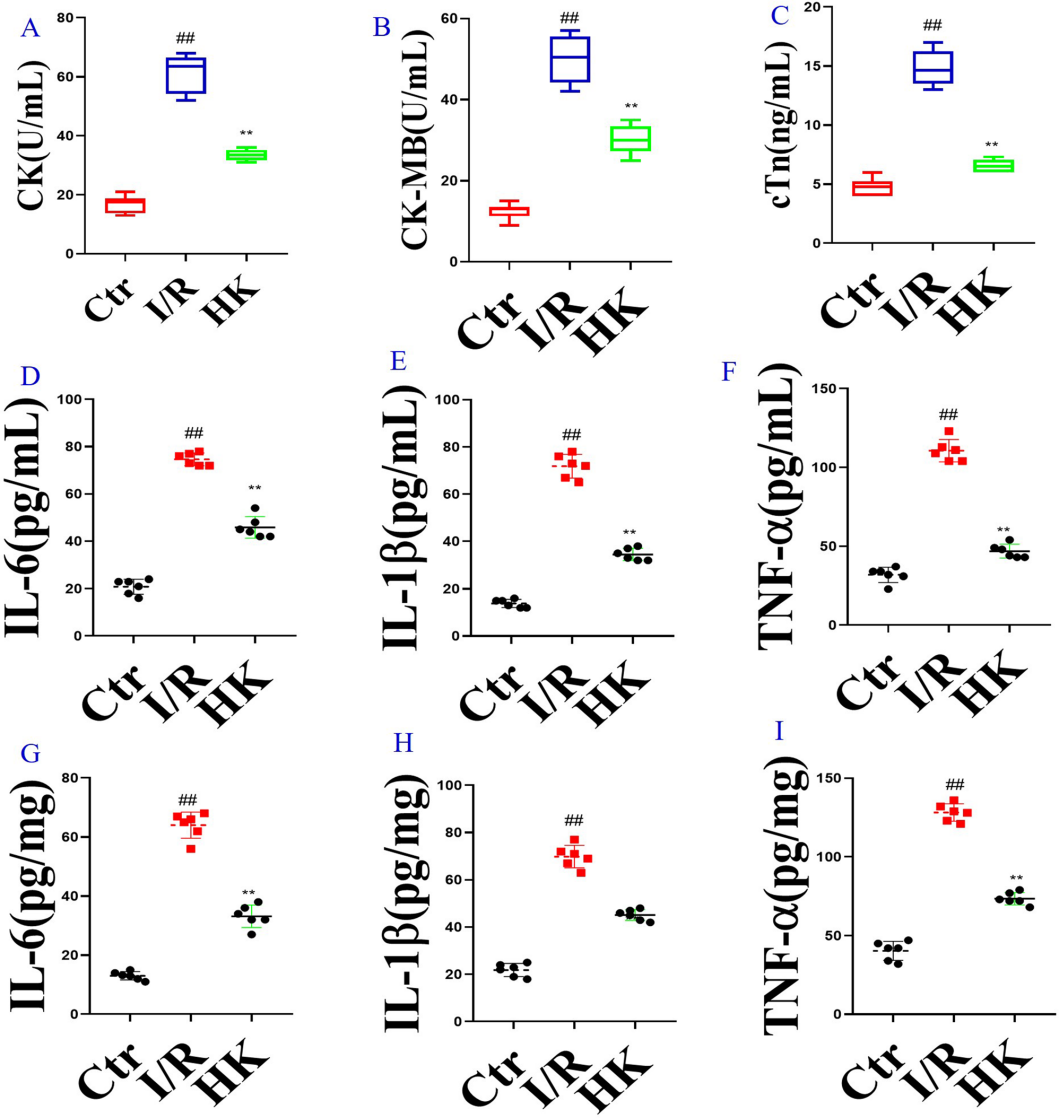
Effects of HMGB3 knockout on biochemical indexes and cytokines in I/R mice. (**A**-**C**) CK, CK-MB, cTn; (**D**-**I**) IL-1β, IL-6, and TNF-α in serum and heart (*n* = 6). All data were presented as mean ± SD. Compared with Ctr: ^##^*P* < 0.01. Compared with I/R: ^**^P < 0.01

**CK**,** CK-MB**,** cTn**,** AST**,** ALT**,** UN and Cr in serum and heart tissues in I/R mice.** As shown in Fig. 3A-I, as compared with sham group (WT mice), the levels of CK, CK-MB, cTn, IL-1β, IL-6 and TNF-α in serum or heart tissues in I/R mice were significantly increased. As compared with I/R mice, the levels of CK, CK-MB, cTn, IL-1β, IL-6 and TNF-α in serum or heart tissues in HMGB3 KO mice were significantly decreased. The results suggest that HMGB3 KO may alleviate the myocardial I/R injury, HMBG3 plays an important role in acute myocardial I/R injury.

**Fig. 4:**
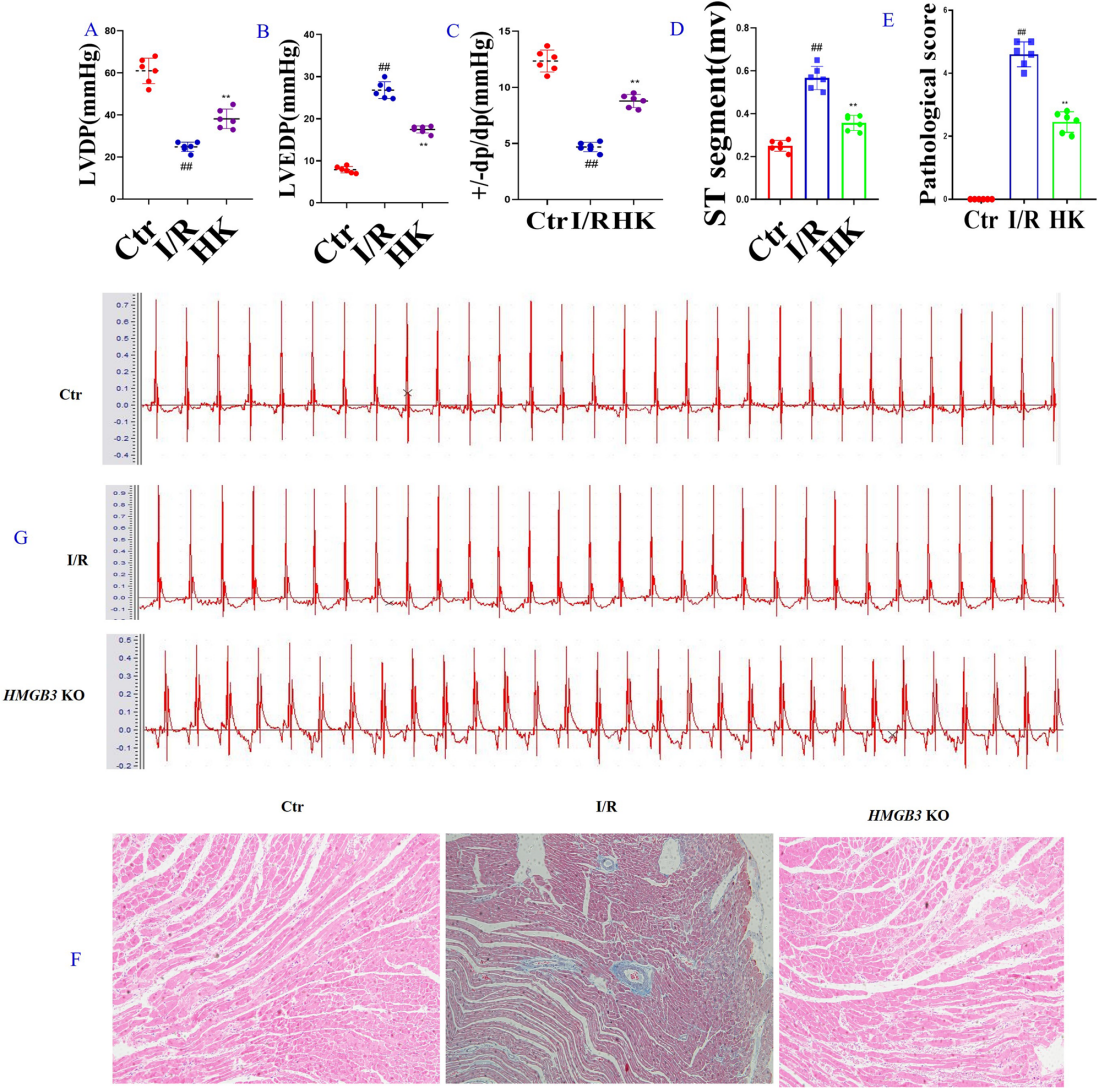
Effects of HMGB3 knockout on cardiac function and cardiac pathology. (**A**-**C**) LVDP, LVEDP and +/-dp/dt; The changes of electrocardiogram (**D**, **F**) and ST segment (**E**, **G**). Pathological examination of I/R mice and pathological score (**G**). (*n* = 6). All data were presented as mean ± SD. Compared with Ctr: ^##^*P* < 0.01. Compared with I/R: ^**^P < 0.01

**Hemodynamics and electrocardiogram in I/R mice.** Hemodynamics plays an important role in myocardial I/R. In this study. Therefore, this experiment examined the change of hemodynamics in I/R mice. As shown in Fig. 4A-C, as compared with sham group (WT mice), the levels of LVDP, +/-dp/dt were significantly decreased and LVEDP were significantly increased in I/R mice. HMGB3 alleviated these changes of LVDP, +/-dp/dt and LVEDP in I/R mice. As for electrocardiogram, the results of electrocardiogram were in Fig. 4D, F. as compared with sham group (WT mice), ST segment significantly elevated in I/R mice. As expected, compared with I/R mice, HMGB3 KO significantly reduced ST segment. The results suggest that HMBG3 plays important role in cardiac dysfunction during acute myocardial ischemic injury.

**Fig. 5:**
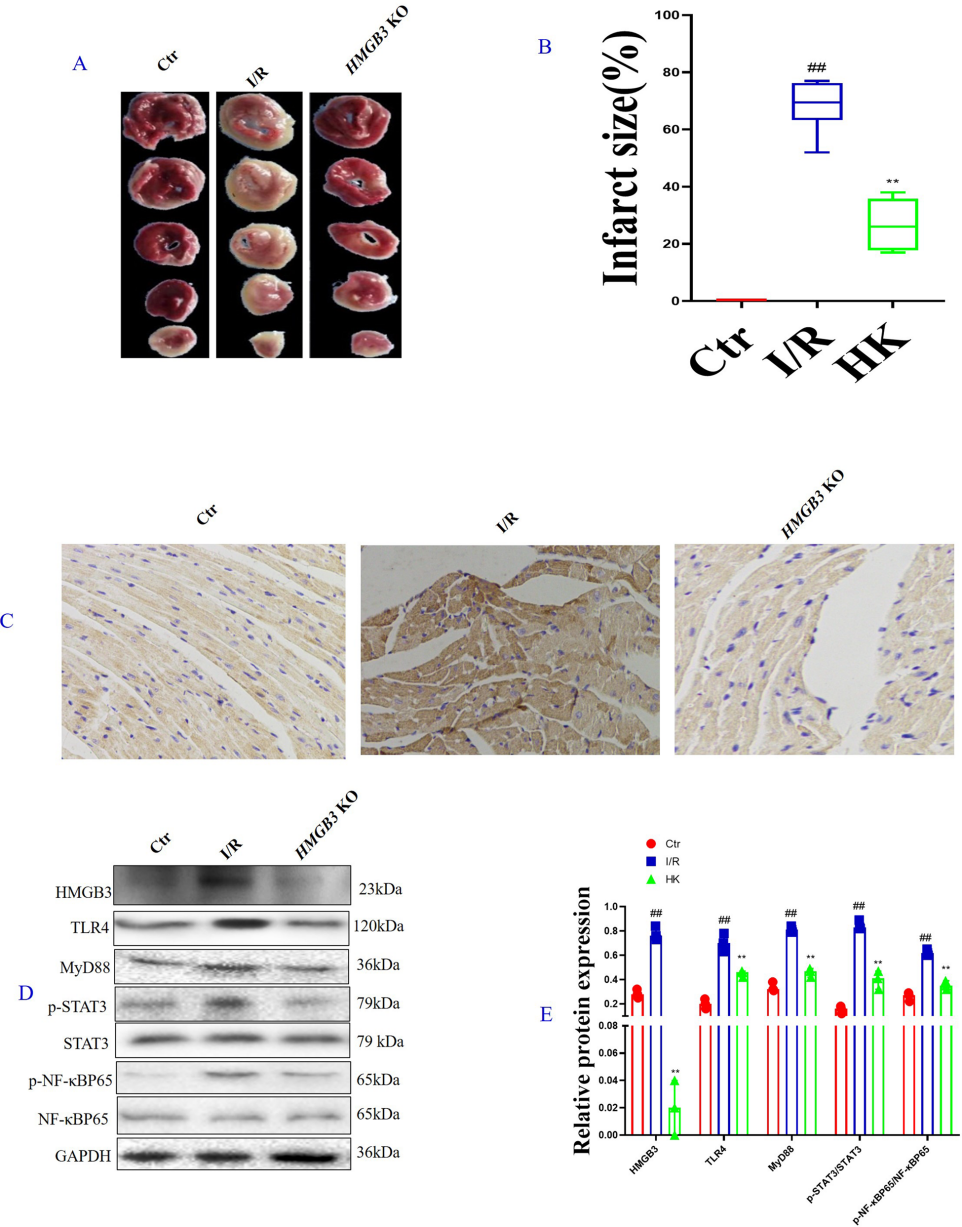
Effects of HMGB3 knockout on myocardial infarction area and related protein expression. (**A**-**B**) TTC staining of heart and infarct size; (**C**) immunohistochemical results of TLR4; (**D**-**E**) The levels of HMGB3, p-STAT3, TLR4, MyD88, and p-NF-κBP65. (*n* = 6). All data were presented as mean ± SD. Compared with Ctr: ^##^*P* < 0.01. Compared with I/R: ^**^P < 0.01

**HE and TTC staining.** Pathological examination is very important for myocardial I/R injury. As shown in Fig. 4E-G, as compared with sham group (WT mice), in I/R group, myocardial cells were obviously edema, myofilaments were disordered, degraded and necrotic in different degrees, and accompanied by inflammatory cell infiltration. HMGB3 KO alleviated the pathological injury of myocardial cells. As for TTC staining, as compared with sham group (WT mice), after TTC staining, white areas where can be seen in the heart of I/R group. At the same time, the myocardial infarction area in I/R group significantly increased. As expected, HMGB3 KO alleviated above changes (Fig. 5A-B).

#### In cell experiments

**Fig. 6:**
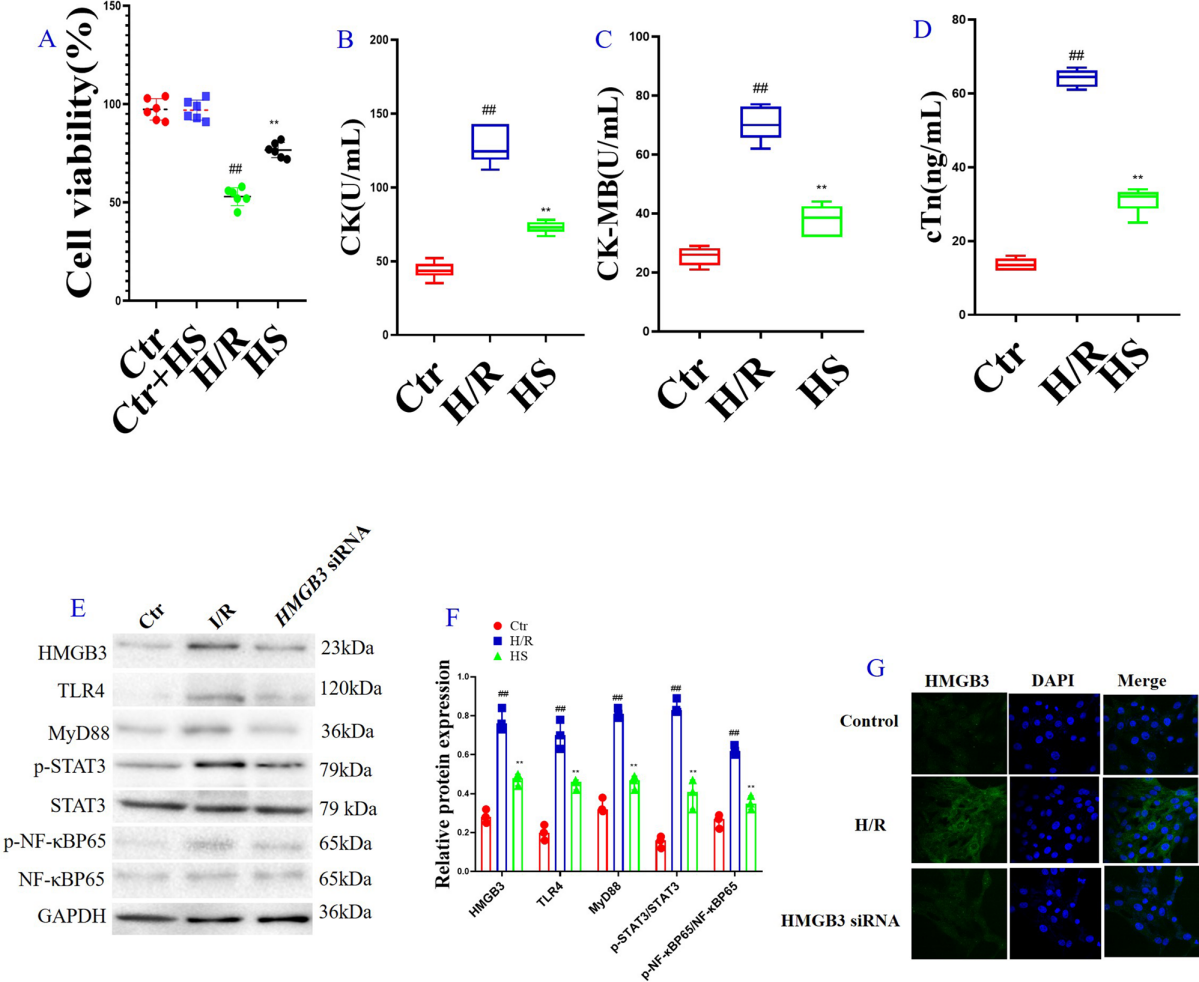
Effects of HMGB3 knockout on H/R-induced H9c2 cells. (**A**) Cell viability of H/R; (**B**-**D**) the levels of CK, CK-MB and cTn(M) in H9c2 cell; (**E**-**F**) The levels of HMGB3, p-STAT3, TLR4, MyD88, and p-NF-κBP65;(**G**) Immunofluorescence of HMGB3. All data were presented as mean ± SD. Compared with Ctrl: ^##^*P* < 0.01. compared with H/R: ^**^P < 0.01

**Cell viability.** As shown in Fig. 6A, as compared with the control group, the cell viability in H/R was significantly decreased. As compared with H/R group, the cell viability in HMGB3 siRNA were significantly increased. The results suggest that HMGB3 plays important role in cell H/R injury.

**CK**,** CK-MB**,** and cTnin H9c2 cells.** As shown in Fig. 6B-D, as compared with the control group, the levels of CK, CK-MB and cTn, in H/R cell were significantly decreased. As compared with H/R group, the levels of CK, CK-MB and cTn, in HMGB3 siRNA groups were significantly decreased.

### The mechanism of HMGB3 in myocardial I/R injury

#### In animal experiments

To study and verify the mechanism of HMGB3 in I/R injury, western blot was used to detect the expression of related proteins. As shown in Fig. 5D-E, as compared with the sham group (WT mice), the levels of HMGB3, p-STAT3, TLR4, MyD88, and p-NF-κBP65 in heart tissues in I/R mice were increased. As compared with I/R mice, the levels of HMGB3, p-STAT3, TLR4, MyD88, and p-NF-κBP65 in heart tissues in HMGB3, TLR4 and STAT3 KO mice were significantly decreased. Immunohistochemistry result were consistent with the western blot results (Fig. 5C). The results showed that HMGB3 mediated myocardial injury was related to TLR4/STAT3 signal pathway.

#### In cell experiments

To study and verify the mechanism of HMGB3 in H/R cell, western blot was used to detect the expression of related proteins. As shown in Fig. 6E-F, as compared with the control group, the levels of HMGB3, p-STAT3, TLR4, MyD88, and p-NF-κBP65 in H/R group were increased. As compared with H/R group, the levels of HMGB3, p-STAT3, TLR4, MyD88, and p-NF-κBP65 in HMGB3 siRNA, TLR4 siRNA and STAT3 siRNA groups were significantly decreased. Immunofluorescence results were consistent with the western blot results (Fig. 6G). The results showed that HMGB3 mediated myocardial cell injury was related to TLR4/STAT3 signal pathway.

**Fig. 7:**
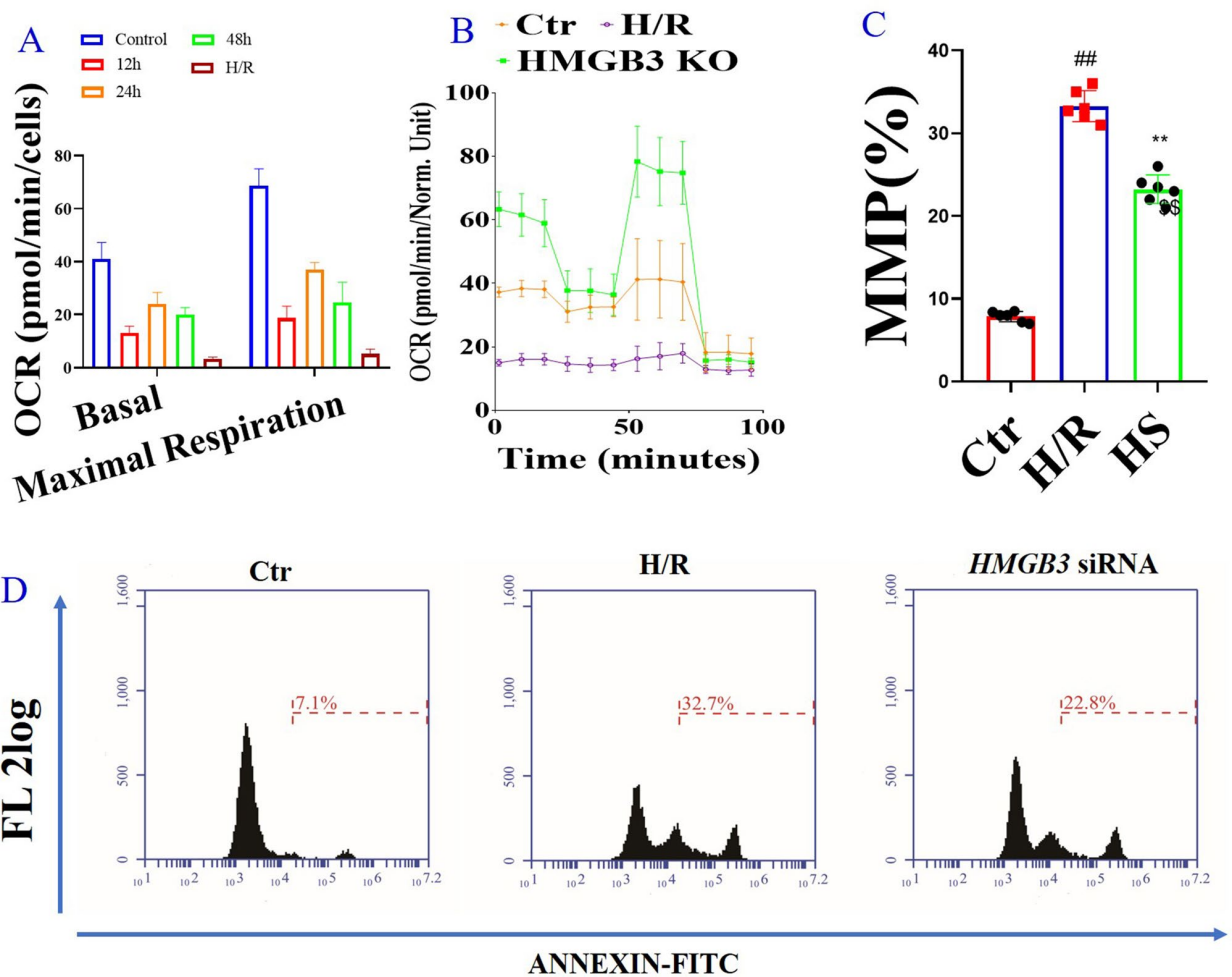
Effects of HMGB3 knockout on mitochondrial function. (**A**-**B**) mitochondrial respiration; (**C**-**D**) mitochondrial membrane potential. All data were presented as mean ± SD. Compared with Ctrl: ^##^*P* < 0.01. compared with H/R: ^**^P < 0.01

Since the overexpression of HMGB3 will shift to mitochondria, because HMGB3 has an effect on mitochondrial function, we tested the effect of HMGB3 knockout on mitochondrial function. The results showed that after HMGB3 knockout, mitochondrial respiration was improved and mitochondrial membrane potential significantly decreased (Fig. 7A-D).

#### The effects of HMGB3 KO (HMGB3 siRNA) and TLR4 inhibitor (TAK-242) or TLR4 siRNA on CK, CK-MB, cytokine in serum, cardiac pathological changes and STAT3 protein expression in I/R mice and H/R-treated H9c2 cells

##### In I/R mice

**Fig. 8:**
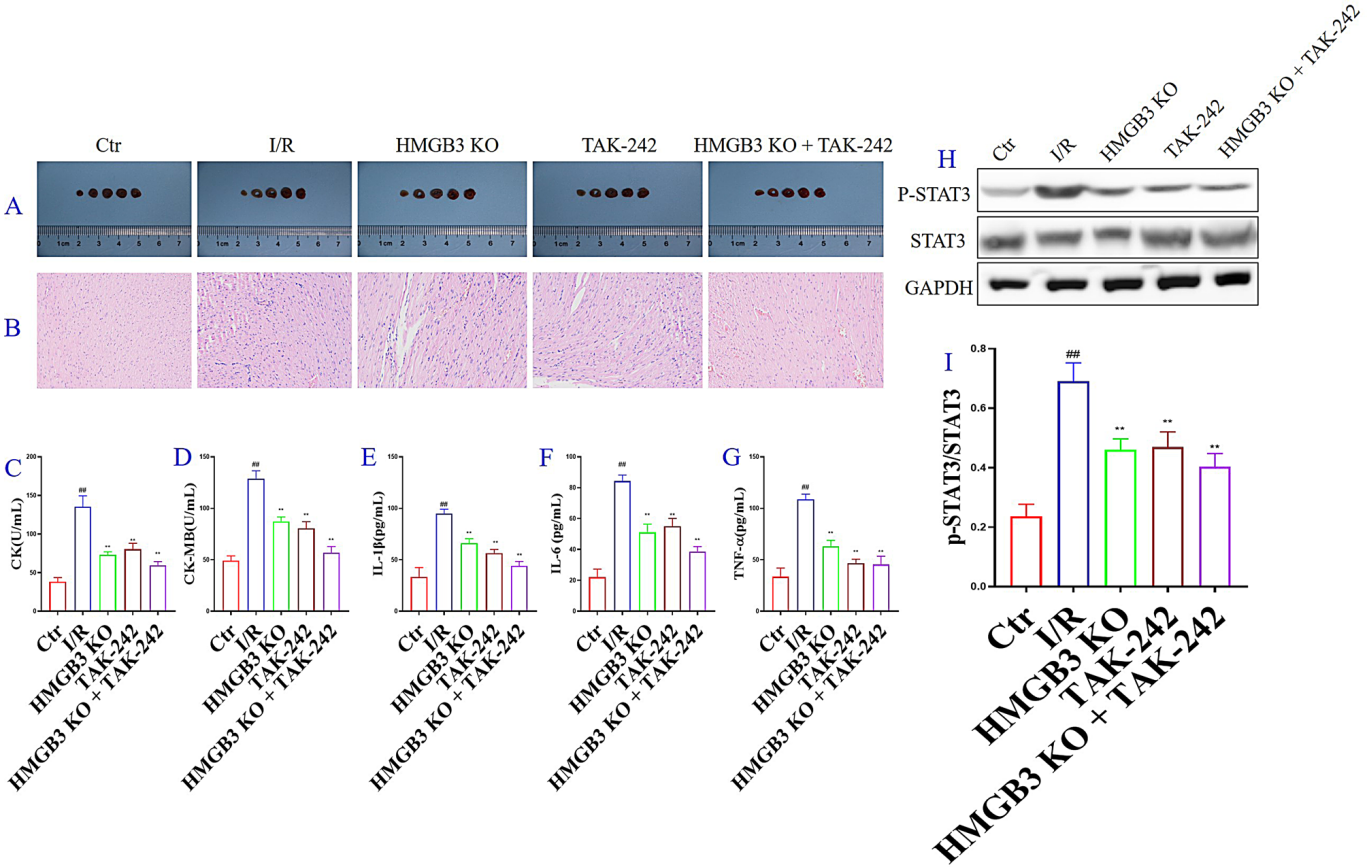
The effects of HMGB3 KO (HMGB3 siRNA) and TLR4 inhibitor (TAK-242) or TLR4 siRNA on CK, CK-MB, cytokine in serum, cardiac pathological changes and STAT3 protein expression in I/R mice. (**A**) TTC staining; (**B**) HE staining; (**C**-**D**) The levels of CK and CK-MB; (**E**-**F**) IL-1β, IL-6, and TNF-α in serum; (**H**-**I**) The level of p-STAT6. All data were presented as mean ± SD. Compared with Ctrl: ^##^*P* < 0.01. compared with I/R: ^**^P < 0.01

As compared with sham group, the levels of CK and CK-MB in serum were increased in I/R group, HMGB KO and TLR4 inhibitor (TAK-242) significantly decreased the levels of CK, CK-MB and cytokine in mice serum. In addition, as compared with HMGB KO and TLR4 inhibitor (TAK-242) group, HMGB3 KO + TLR4 inhibitor (TAK-242) significantly decreased the levels of CK, CK-MB and cytokine in mice serum. As compared with sham group, in I/R group, myocardial cells presented obvious edema, the myofilaments were disordered, degraded and necrotic in different degrees, and accompanied by inflammatory cell infiltration. HMGB3 KO and TLR4 inhibitor (TAK-242) alleviated the pathological injury of myocardial cells. In addition, as compared with HMGB KO and TLR4 inhibitor (TAK-242) group, HMGB3 KO + TLR4 inhibitor (TAK-242) significantly restored the changes of pathological injury of myocardial cells. As for TTC staining, as compared with sham group (WT mice), white areas were seen in the heart in I/R group. At the same time, the myocardial infarction area in I/R group was significantly increased. As expected, HMGB3 KO and TAK-24 group alleviated the changes. In addition, HMGB3 KO + TAK-24 group more significantly restored these changes. As compared with sham group, the level of p-STAT3 in heart tissues were increased in I/R group, HMGB KO and TLR4 inhibitor (TAK-242) significantly decreased the level of p- STAT3 in heart. In addition, as compared with HMGB KO and TLR4 inhibitor (TAK-242) group, HMGB3 KO + TLR4 inhibitor (TAK-242) further decreased the expression level of p-STAT3 in heart tissue of I/R mice (Fig. 8A-H).

##### H/R-treated H9c2 cells

As shown in Fig. 4E-G, as compared with sham group, the levels of CK and CK-MB in H/R-treated H9c2 cells were increased, HMGB3 siRNA and TLR4 siRNA significantly decreased the levels of CK, CK-MB and cytokine in H/R-treated H9c2 cells. In addition, as compared with HMGB3 siRNA and TLR4 siRNA groups, HMGB3 siRNA + TLR4 inhibitor (TAK-242) further decreased the levels of CK, CK-MB and cytokine in H/R-treated H9c2 cells.

**Fig. 9:**
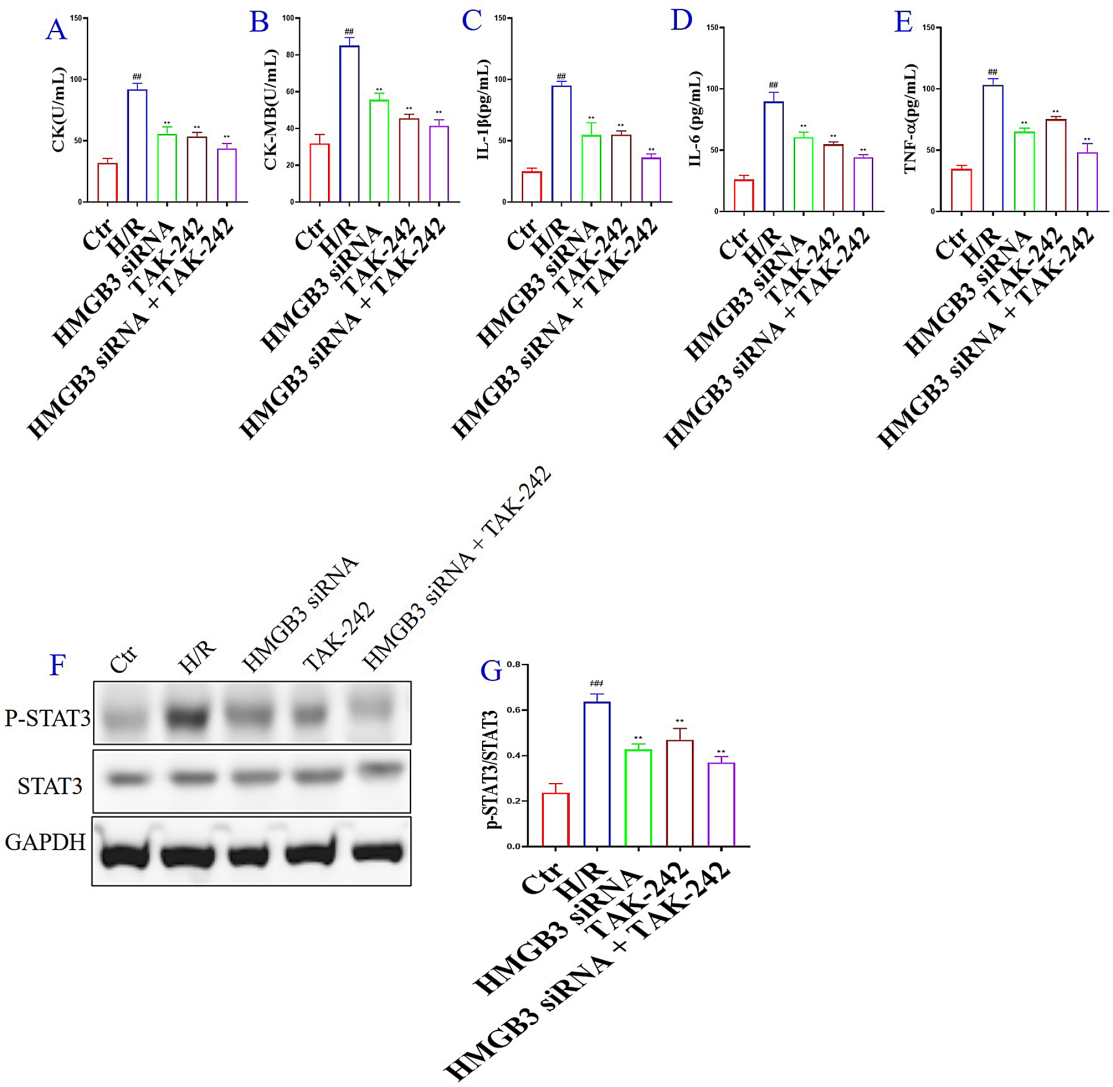
The effects of HMGB3 KO (HMGB3 siRNA) and TLR4 inhibitor (TAK-242) or TLR4 siRNA on CK, CK-MB, cytokine in serum, cardiac pathological changes and STAT3 protein expression in H/R H9c2 cells. (**A**-**B**) the levels of CK and CK-MB; (**C**-**E**) IL-1β, IL-6, and TNF-α in serum; (**F**-**G**) the level of p-STAT6

As shown in Fig. 4E-G, as compared with sham group, the level of p- STAT3 in H/R-treated H9c2 cells were increased in I/R group, HMGB siRNA and TLR4 siRNA significantly decreased the level of p-STAT3 in H/R-treated H9c2 cells. In addition, as compared with HMGB siRNA and TLR4 siRNA group, HMGB3 siRNA + TLR4 siRNA further decreased the expression level of p-STAT3 in H/R-treated H9c2 cells (Fig. 9A-G).

#### Interaction between HMGB3 and TLR4

**Fig. 10:**
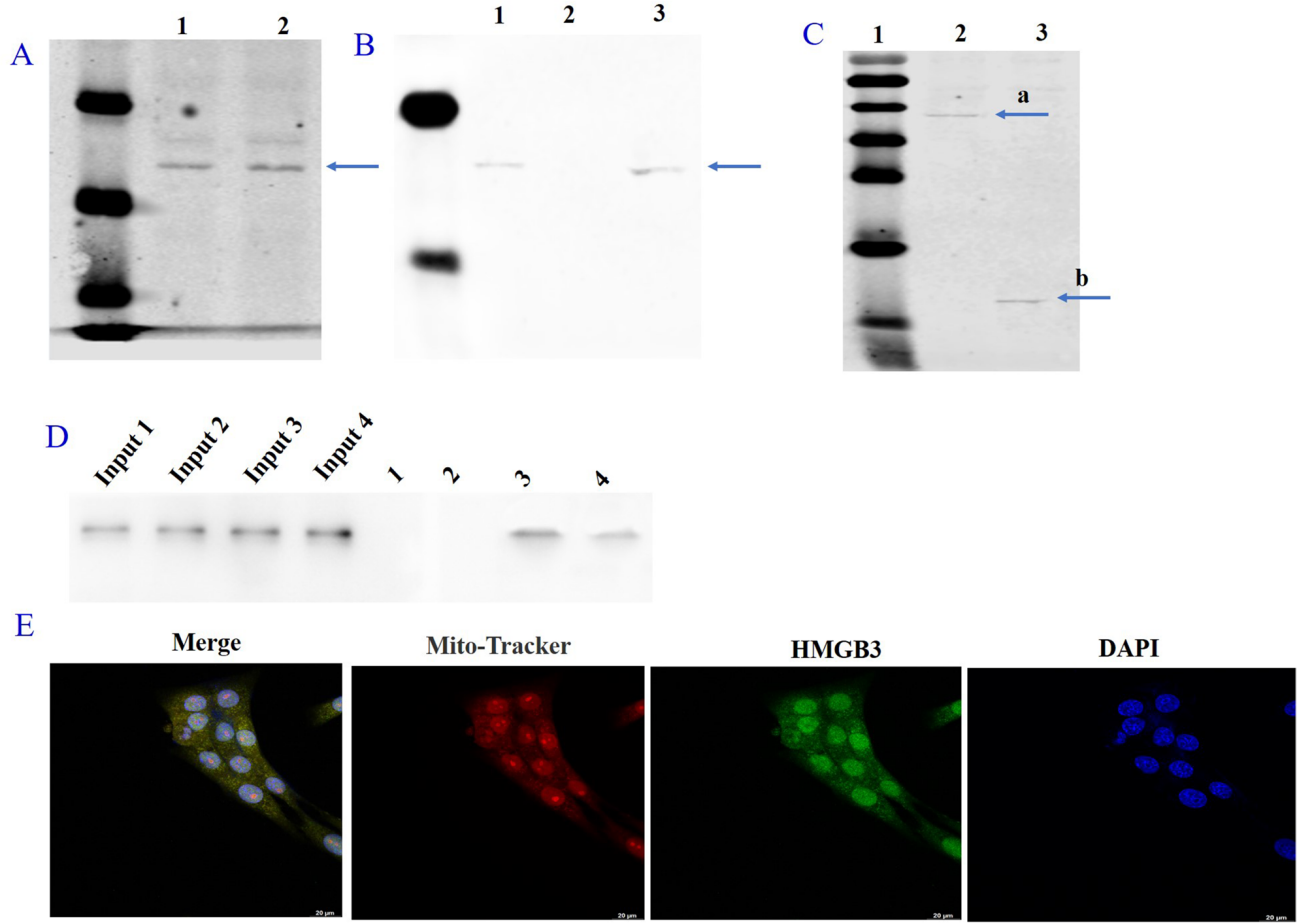
Interaction between HMGB3 and TLR4. (**A**)Results of in vitro translation and identification of pET28a (+) – HMGB3 recombinant plasmid: 1, 2 His-HMGB3 fusion protein; (**B**) Identification results of purified GST-TLR4 fusion protein by Coomassie brilliant blue staining: 1-Marker, 2- GST-TLR4 fusion protein,3-GST protein. a: GST-TLR4 fusion protein, b: GST protein; (**C**) Direct binding of GST-TLR4 and His-HMGB3 fusion protein in vitro. 1: His-HMGB3 control; 2: Negative control; 3: His-HMGB3 protein bands in binding products; (**D**) Mitochondrial localization of HMGB3

**Identification of recombinant His-HMGB3 and GST-TLR4 proteins.** After pET28a (+)-HMGB3 recombinant plasmid was constructed and identified. The recombinant plasmid was translated in vitro, and the product was proceeded SDS-PAGE electrophoresis, gel extraction and tablet pressing after electrophoresis, and autoradiographic detection (Fig. 10A). After pGEX-KG-TLR4 recombinant plasmid was constructed and identified, it was expressed in large quantities. Transforming pGEX-KG-TLR4 recombinant plasmid into DH5α E. coli was incubated at 37 ℃ and then lysed for protein purification, followed by Western blot and test staining.

**Results of GST pulldown test of GST-TLR4 and His-HMGB3 fusion protein.** After the purified protein of GST-TLR4 and the in vitro translation product of His-HMGB3 were obtained, the GST pulldown experiment of the two was further carried out. They were divided into two groups, one of which was a negative control. The purified GST protein was used for binding reaction with His-HMGB3. The results showed that there was radioactive protein in the binding reaction product, and the position of the electrophoretic band was consistent with that of His-HMGB3, indicating that the full length of His-HMGB3 and GST-TLR4 could be directly bound in vitro, but there was no binding band with GST protein, which further demonstrated the specificity of the binding reaction (Fig. 10B).

**HMGB3 and TLR4 proteins can bind to each other in cells.** The results of electrophoresis showed that input1-4 had bands, indicating that HMGB3 protein was expressed in cells of each group, and the expression level in cells of each group was consistent, which provided a strong comparability for the results of coprecipitation experiment. Group 1 and group 2 have no bands, group 3 has weak bands, and group 4 has bands. It indicated that HMGB3 and flag-tag protein cannot bind and form complex. Flag beads can only precipitate flag-tag protein, but not HMGB3 protein from cell lysate. Therefore, no positive band can be detected when anti HMGB3 antibody was used for Western blot analysis. In groups 3 and 4, the cells expressed flag-TLR4 fusion protein. TLR4 and HMGB3 could combine to form a complex. Therefore, HMGB3 bands could be detected in the precipitated protein. The band of group 4 was stronger, indicating that the application of PMA + ionomycin stimulation could enhance the binding between HMGB3 and TLR4. The application of flag beads could precipitate more HMGB3 protein, and the detected band was more concentrated. The results of cell localization were consistent with the above results (Fig. 10C-D). Meanwhile, immunofluorescence showed that HMGB3 colocalized with mitochondria (Fig. 10E).

### The protective effect of mitochondrial targeted HMGB3 siRNA (TPP-PEI/siRNA) on myocardial I/R injury

**Fig. 11:**
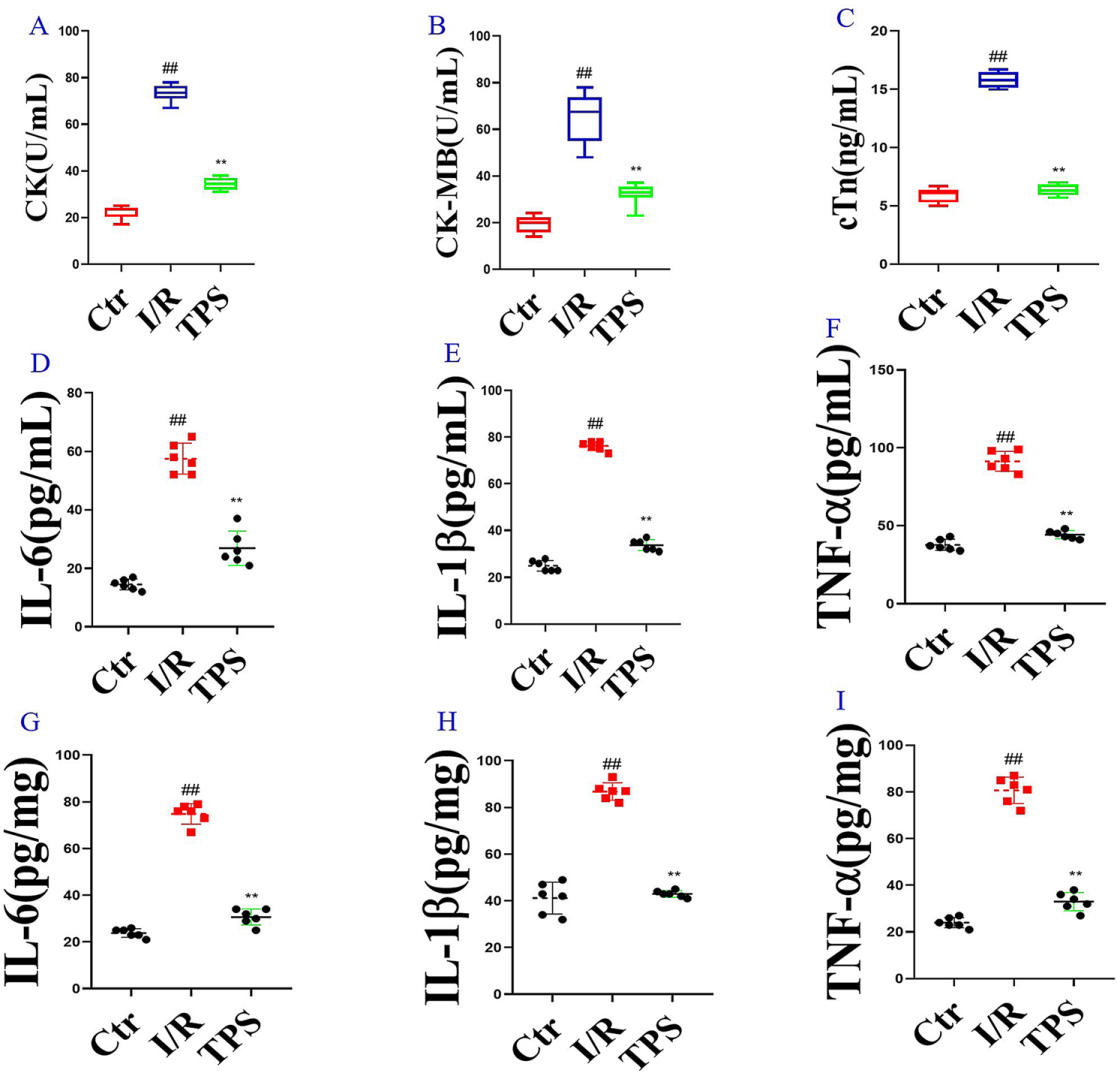
Effects of TPP-PEI/HMGB3 siRNA (TPS) on biochemical indexes and cytokines in I/R mice. (**A**-**C**) CK, CK-MB, cTn; (**D**-**I**) IL-1β, IL-6, and TNF-α in serum and heart (*n* = 6). All data were presented as mean ± SD. Compared with Ctr: ^##^*P* < 0.01. Compared with I/R: ^**^P < 0.01

**Fig. 12:**
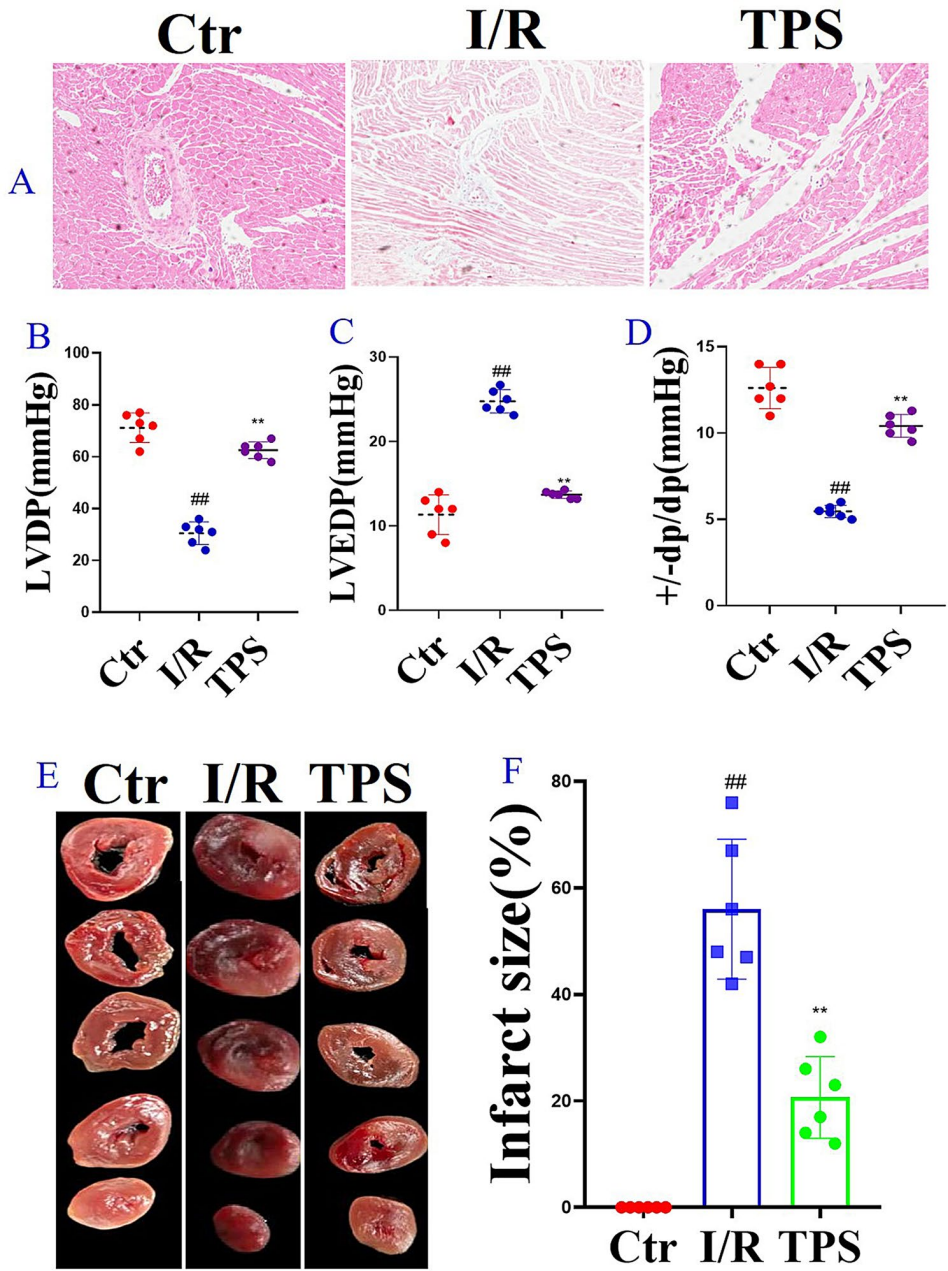
Effects of TPP-PEI/HMGB3 siRNA (TPS) on cardiac function and cardiac pathology and myocardial infarction area. (**A**) Hematoxylin-eosin (HE) staining examination of I/R mice; (**B**-**D**) LVDP, LVEDP and +/-dp/dt; (**E**-**F**) TTC staining of heart and infarct size. Compared with Ctr: ^##^*P* < 0.01. Compared with I/R: ^**^P < 0.01

In order to further confirm the role of HMGB3 in acute myocardial ischemia injury and understand the protective effect of mitochondrial targeted HMGB3, we observed the protective role of a newly mitochondrial targeted HMGB3 siRNA (TPP-PEI/siRNA, TPS) in myocardial I/R mice and H/R H9c2. In I/R mice, the results showed that TPP-PEI/siRNA polymer significantly decreased the levels of CK, CK-MB, cTn, IL-1β, IL-6 and TNF-α in serum and heart tissues of I/R mice, improved the hemodynamics and histopathological index of I/R mice. This was because HMGB3 was knocked out, which led to disappearance of the targeting effect of TPP-PEI/siRNA polymer (Figs. 11A-I and 12A-F).

**Fig. 13:**
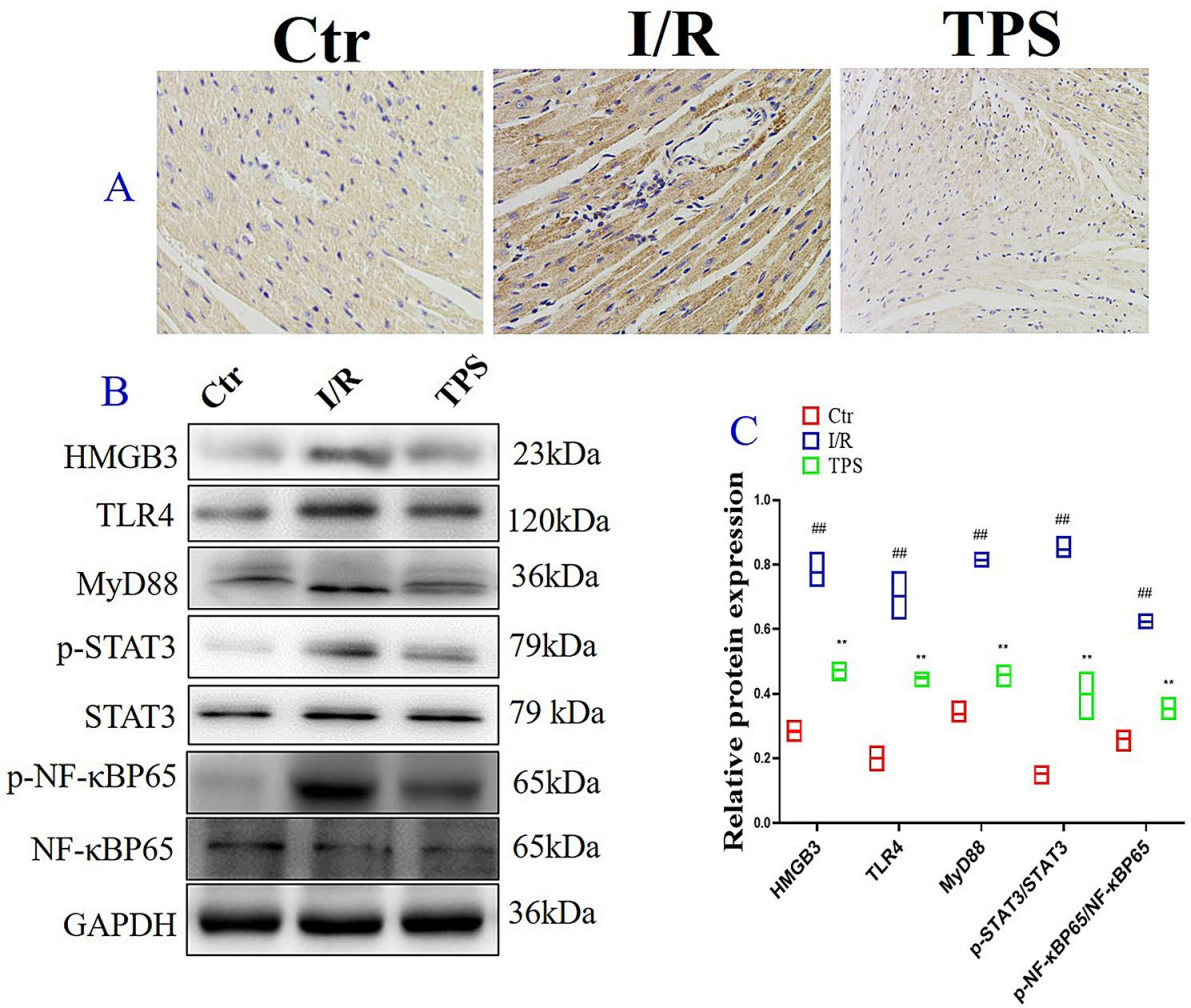
Effects of TPP-PEI/HMGB3 siRNA (TPS) on H/R-induced H9c2 cells. (**A**-**C**) CK, CK-MB, cTn; (**D**-**F**) IL-1β, IL-6, and TNF-α in H9c2 cells (*n* = 6). All data were presented as mean ± SD. Compared with Ctr: ^##^*P* < 0.01. Compared with H/R: ^**^P < 0.01

In H/R, the results showed that TPP-PEI/siRNA alleviated HMGB3 mediated myocardial cell injury. The cell viability was significantly increased, CK, CK-MB and cTn was significantly decreased and the cell pathology was significantly improved in H/R + TPP-PEI/siRNA group. These results suggest that mitochondrial targeting HMGB3 siRNA has good protective effect on myocardial I/R injury, HMGB3 is an important target for the treatment of acute myocardial ischemic injury (Fig. 13A-F).

**Fig. 14:**
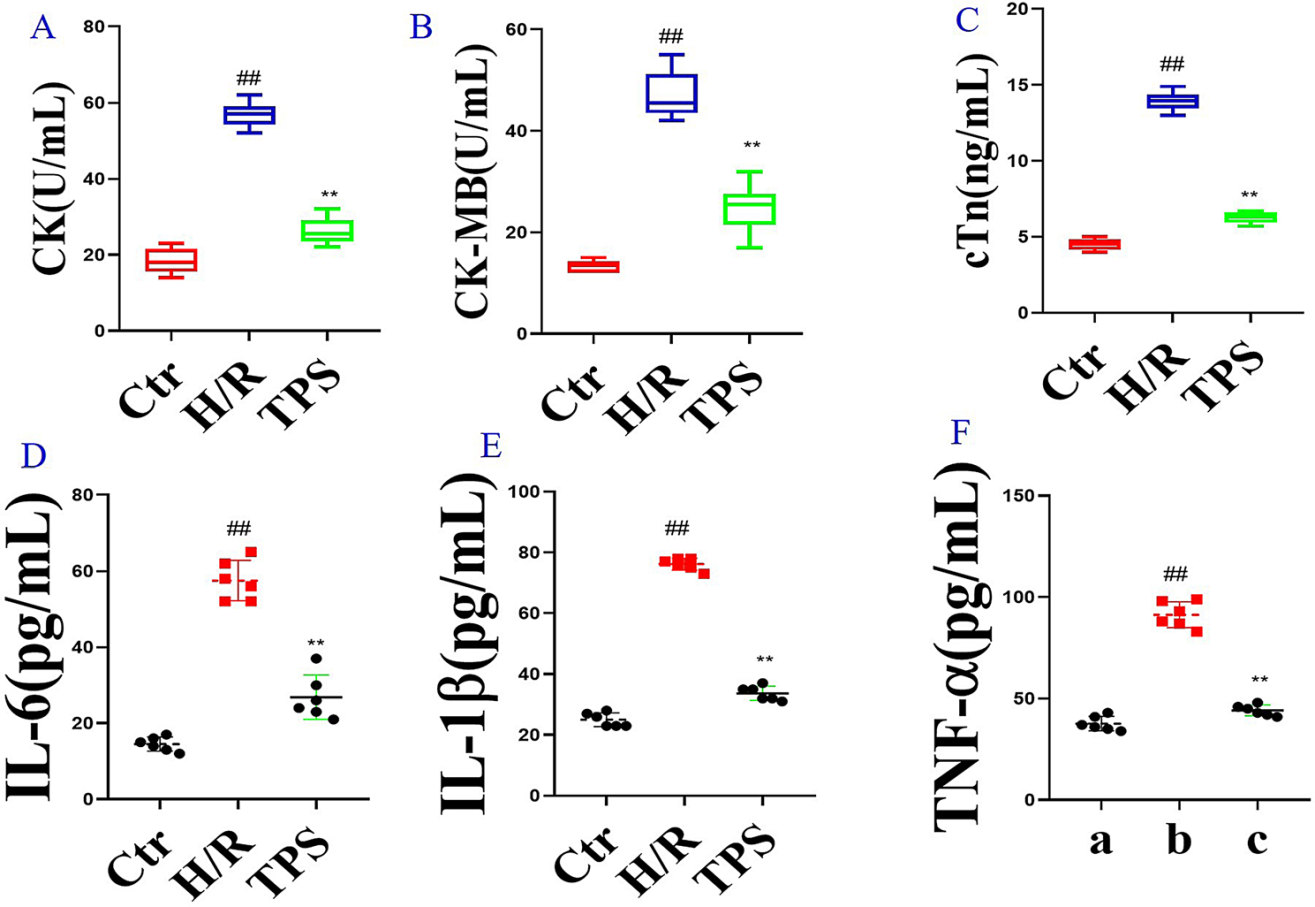
Effects of TPP-PEI/HMGB3 siRNA (TPS) on HMGB3-related protein expression in mice. (**A**) Immunohistochemical results of TLR4; (**B**-**C**) The levels of HMGB3, p-STAT3, TLR4, MyD88, and p-NF-κBP65. Compared with Ctr: ^##^*P* < 0.01. Compared with I/R: ^**^P < 0.01

**Fig. 15:**
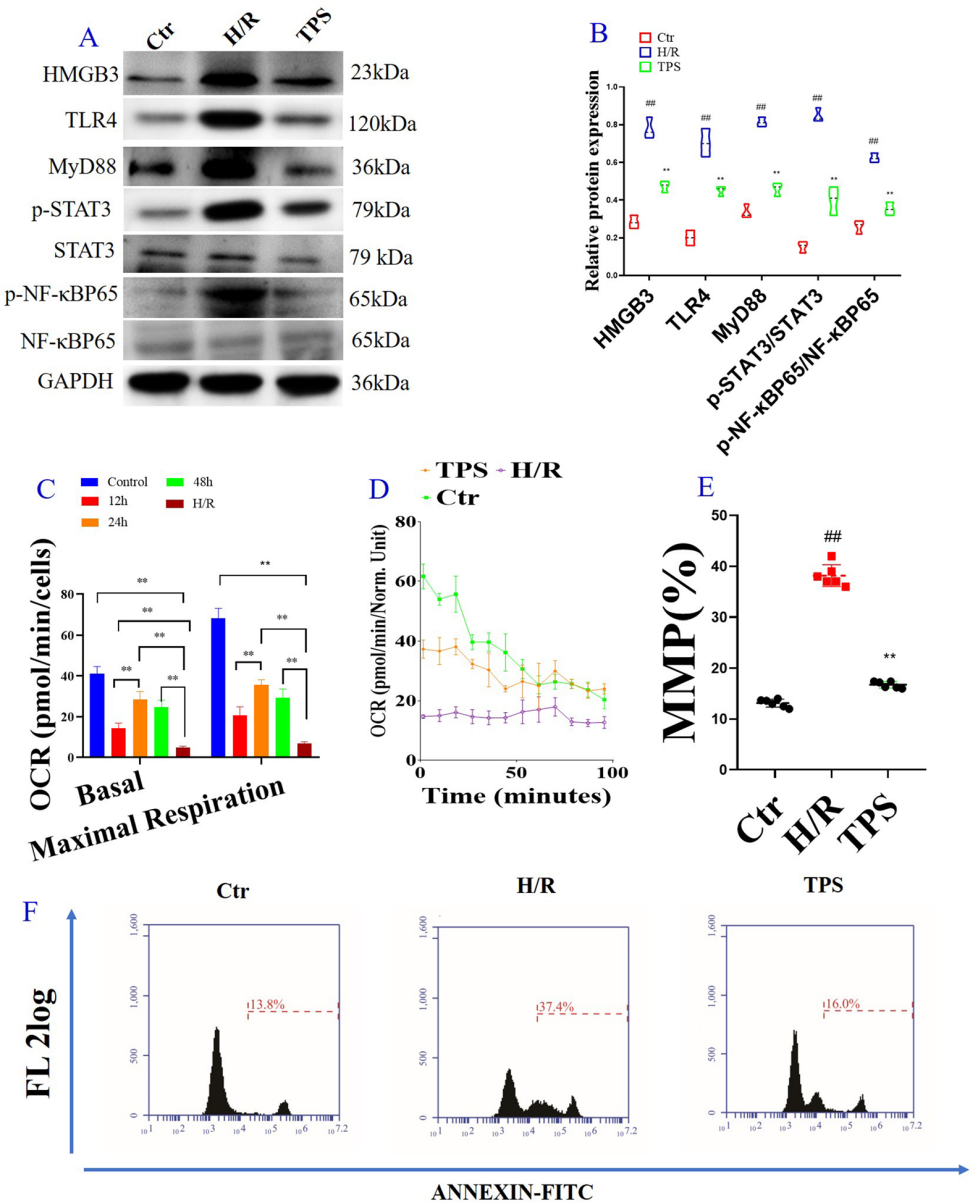
Effects of TPP-PEI/HMGB3 siRNA (TPS) on HMGB3-related protein expression and mitochondrial function. (**A**-**B**) The levels of HMGB3, p-STAT3, TLR4, MyD88, and p-NF-κBP65; (**C**-**D**) mitochondrial respiration; (**E**-**F**) mitochondrial membrane potential. Compared with Ctr: ^##^*P* < 0.01. Compared with H/R: ^**^P < 0.01

The further study found TPP-PEI/siRNA reduced the expression of HMGB3 related signal molecule including p-STAT3, TLR4, MyD88, and p- NF-κBP65 in heart tissues of I/R mice and H/R H9c2 cell. The results further proved that p-STAT3/TLR4 signal pathway participated in HMGB3 mediated acute myocardial ischemia injury (Figs. 14A-C and 15A-B).

Similarly, at the cellular level, the effects of TPS targeted inhibition of hmgb3 on mitochondrial function were examined, we tested the effect of HMGB3 knockout on mitochondrial function. The results showed that after HMGB3 knockout, mitochondrial respiration was improved and mitochondrial membrane potential significantly decreased (Fig. 15C-F).

## Discussion

I/R is the main cause of heart failure after myocardial infarction or cardiac surgery, which is characterized by high morbidity and mortality [10]. Restoration of coronary blood flow rapidly is the only effective way to reduce myocardial injury, but the cardiac I/R itself will also lead to the death of other tissue cells and further expand the myocardial infarction area. The main factors leading to myocardial I/R injury include oxidative stress, inflammatory reaction, and apoptosis. Mitochondria, as the main place for cell respiration and energy metabolism, are the most important Organelle of myocardial cells. But mitochondria are susceptible to oxygen free radical attacks and are the main subcellular targets of ischemic damage, damage to mitochondrial structure and function leading to apoptosis and necrosis in ischemic regions is the most direct cause of exacerbation of AMI damage, so protecting the integrity of mitochondrial structure and function is crucial for delaying the progression of AMI disease [11–13]. In this study, 4628 proteins were identified via Homo sapiens database by proteomics technology with clinical myocardial infarction heart samples. 365 kinds of differentially expressed proteins were identified, and found HMGB3, a high mobility protein, which can combine with mitochondrial DNA (mDNA), was found to have the greatest expression change in MIT. With myocardial I/R and HMGB3, TLR4 and STAT3 KO mice and H/R H9c2 cells, we found HMGB3 played an important role in acute myocardial ischemic injury. Mitochondrial targeted HMGB3 siRNA antagonized the role of HMGB3 and played a good protective effect for myocardial I/R injury.

High mobility group-box3 (HMGB3) belongs to the high mobility group protein family and plays an important role in DNA replication and transcription [14]. HMGB is a good ultimate killer because of its combination with DNA and mitochondria [15–16]. Three different HMGB subtypes, namely HMGB1, HMGB2 and HMGB3, have been identified with 80% sequence identity in humans. HMGB1, HMGB2 and HMGB3 are also the important cytokines that mediate responses to infection, injury, and inflammation, and are called “nuclear weapons” in vertebrate immune library [17]. The main research of HMGB3 focuses on the pathophysiological of tumor such as cervical cancer, lung cancer [18]. But there are few reports on heart disease especially for acute myocardial ischemia such as AMI. In this study, we analyzed the difference of proteins between infarcted tissues and non-infarcted tissues in AMI patients by proteomics technique, and found out the differential protein HMGB3. To verify the pathophysiological role of HMGB3 in AMI, in this experiment, the interference and knock-out experiments of HMGB3 were carried out in vivo and in vitro. The experimental results showed that interference and knock-out of HMGB3 significantly alleviated acute myocardial ischemic injury. It is suggested that HMGB3 plays an important role in acute myocardial ischemic injury.

What is the mechanism of HMGB3 that mediates myocardial ischemic injury? It is not clear. In order to elucidate this issue, the signal pathways mediated by HMGB3 in acute myocardial ischemia were studied in this experiment. Under inflammatory conditions, TLR signaling pathway can be activated by IL-1β/high mobility group box (HMGB) [19]. Toll-like receptors (TLRs) are a kind of pathogen pattern recognition receptors, which activate to release a variety of cytokines by activating nonspecific and specific immunity, and play an important role in various inflammatory reactions [20]. After TLRs can be activated by external factors, TLRs can mediate inflammatory reaction and release cytokines such as IL-6 and then cytokines can combine with their receptors to activate STAT3 [21]. In this study, after interference or knock-out of HMGB3, TLR4 and STAT3, the myocardial ischemia-reperfusion injury was significantly alleviated both in vivo and in vitro. Further, in order to further verify the role of HMGB3 mediated TLR4/STAT3 signaling pathway in I/R and H/R signaling, we further interfered with TLR4 and suppressed TLR4 expression, and detected changes in related indicators. The results showed that further inhibition or interference with TLR4 significantly reduced the levels of CK, CK-MB, improved pathological changes, and decreased p-STAT3 expression. The results suggested that HMGB3 mediated I/R and H/R myocardial injury was related to TLR/STAT3 signal pathway.

Mitochondria acts as the energy “pump” of human body, providing energy support for the survival and development of living organisms continuously. HMGB3 can enter mitochondria and combine with mitochondrial DNA (mDNA) to interfere with the mitochondrial function when its expression is increased. Mitochondrial targeted intervention has attracted more and more attention in many diseases [22]. In the present study, a mitochondrial targeted material TPP-COOH was designed and linked with PEI to form TPP-PEI polymer, which has the function of carrying siRNA. It was found that TPP-PEI-siRNA polymer can target to the myocardial mitochondria and directly intervened the role HMGB3. Therefore, this result suggests that enhancing endogenous antioxidant capacity may be a more effective way to intervene in AMI, especially targeting mitochondrial antioxidant pathways.

In conclusion, HMGB3 is a key protein marker for acute myocardial ischemia, which plays important role in myocardial ischemic injury. The mechanism is mainly related to mitochondrial homeostasis and TLR4/STAT4 signaling pathway. Targeted interfering with mitochondrial HMGB3 with TPP-PET siRNA may treat acute myocardial ischemic injury. This finding provides a new intervention strategy for the treatment of acute myocardial ischemia.

## Supplementary Information

Below is the link to the electronic supplementary material.

Supplementary Material 1

## Data Availability

Data are however available from the corresponding author upon reasonable request and with permission of the corresponding author.

## Abbreviations

MI: Myocardial infarction
DEPs: Differential expressed proteins
MIT: Myocardial infarction
MNIT: Non-infarction tissue
H/R: Hypoxia/reoxygenation
HMGB3: High mobility group protein B3
mDNA: Mitochondrial DNA
TPP: Triphenylphosphine
I/R: Ischemia reperfusion
AMI: Acute myocardial infarction
TNF-α: Tumor necrosis factor (TNF)-α
IL-6: Interleukin (IL)-6
ELISA: Enzyme linked immunosorbent assay
CSO: Color sonography (CSO)
ECG: Electrocardiogram. cTn: Cardiac troponin
CK: Creatine kinase
CK-MB: Creatine kinase isoenzymes
ALT: Serum alanine transaminase
AST: Aspartate aminotransferase
Cr: Creatinine (Cr)
UN: Urea nitrogen
LVEDD: Left ventricular end diastolic diameter
LVEF: Left ventricular ejection fraction
CO: Cardiac output
TPP-COOH: Carboxyamyl triphenyl bromide scale
PEI: Polymine
CLSM: Projection electron microscope
HR: Heart rate
TTC: 2, 3, 5-triphenyltetrazole chloride
