# Supplementary material for: Targeting inhibition of HMGB3 protein transposition in cardiac mitochondria to improve myocardial ischemia reperfusion injury
Source: J Transl Med. 2026 May 20;24:916. doi: 10.1186/s12967-026-07920-w (PMC13374140; doi:10.1186/s12967-026-07920-w)
Supplement: Supplementary file 1 — Supplementary Material 1 [file 12967_2026_7920_MOESM1_ESM.docx]

**Fig.1S TPP-PEI synthesis and siRNA linkage.**

Chematic diagram of TPP-COOH synthesis (A), and TPP-PEI (B). Schematic diagram of linkage between TPP-PEI and siRNA.

**Fig.2S Structural phenotype results of TTP-PEI-mitochondrial targeting siRNA**

Hydrogen spectrum result of TPP-COOH and PEI (A), infrared spectral characteristic of TPP-COOH (B), gel migration retardation result of TPP-PEI-siRNA (C), stability result of TPP-PEI-siRNA (D, E), The effects of TPP-PEI-siRNA on viability of H9c2 cells (F) and d hemolytic reaction (G), the reaction of TPP-PEI/siRNA polymer to pH value (H) and gel retardation results of TPP-PEI/siRNA under different pH conditions (I) and electron microscope result of TPP-PEI/siRNA in pH=4.5 and pH=7.4.
